# FAIRTraits: An enriched, FAIR‐compliant database of plant traits from Mediterranean populations of 240 species

**DOI:** 10.1002/ecy.70219

**Published:** 2025-09-24

**Authors:** Éric Garnier, Léo Delalandre, Jules Segrestin, Karim Barkaoui, Elena Kazakou, Marie‐Laure Navas, Denis Vile, Cyrille Violle, Maud Bernard‐Verdier, Marine Birouste, Alain Blanchard, Iris Bumb, Pablo Cruz, Sandrine Debain, Adeline Fayolle, Claire Fortunel, Karl Grigulis, Gérard Laurent, Sandra Lavorel, Francisco Lloret, Ignacio M. Pérez‐Ramos, Iván Prieto, Catherine Roumet

**Affiliations:** ^1^ CEFE, Univ Montpellier, CNRS, EPHE, IRD Montpellier France; ^2^ Department of Botany, Faculty of Science University of South Bohemia České Budějovice Czech Republic; ^3^ CIRAD, UMR AMAP Montpellier France; ^4^ AMAP, Univ Montpellier, CIRAD, CNRS, INRAE, IRD Montpellier France; ^5^ CEFE, Univ Montpellier, CNRS, EPHE, IRD, Institut Agro Montpellier France; ^6^ LEPSE, Univ Montpellier, INRAE, Institut Agro Montpellier France; ^7^ Centre d'Ecologie et des Sciences de la Conservation (CESCO), Sorbonne Université‐MNHN‐CNRS Paris France; ^8^ INRA‐AGIR, UMR1248 Castanet Tolosan France; ^9^ Forêts et Sociétés, Univ Montpellier, CIRAD Montpellier Montpellier France; ^10^ CIRAD Forêts et Sociétés Libreville Gabon; ^11^ Laboratoire d'Ecologie Alpine Université Grenoble Alpes, Université Savoie Mont‐Blanc, CNRS Grenoble France; ^12^ CREAF Cerdanyola del Vallès Spain; ^13^ Universitat Autònoma de Barcelona Cerdanyola del Vallès Spain; ^14^ Instituto de Recursos Naturales y Agrobiología de Sevilla (IRNAS‐CSIC) Sevilla Spain; ^15^ Área de Ecología, Facultad de Ciencias Biológicas y Ambientales, Departamento de Biodiversidad y Gestión Ambiental Universidad de León León Spain

**Keywords:** biodiversity standards, ecosystem properties, environmental conditions, FAIR principles, gas exchange, leaf, stem, root and reproductive traits, litter mass loss, Mediterranean Biogeographic Region, phenology, plant functional traits, terminological resources, trait‐based ecology

## Abstract

Trait‐based ecology relies on high‐quality, well‐documented data to explore how plant traits relate to environmental conditions, community assembly, and ecosystem functioning. However, the reuse and synthesis of trait data across studies remain limited by several constraints: a lack of detailed metadata, heterogeneous protocols, absence of individual‐level measurements, and underrepresentation of certain trait types—particularly below‐ground traits. Many existing datasets also lack the environmental details necessary to investigate trait–environment relationships at local scales. Here, we present FAIRTraits, a comprehensive dataset that addresses these limitations by compiling 189,452 records of quantitative trait measurements collected between 1997 and 2023 from 1955 populations of 240 vascular plant species in the Northern Mediterranean Basin, a region known both for its exceptional biodiversity and as a climate change hotspot. All data were collected by a single research group using consistent and well‐documented field and laboratory protocols, ensuring internal consistency across traits, species, sites, and years. FAIRTraits includes 180 traits measured at the individual or replicate level, with no aggregation. It features an unprecedented diversity of traits spanning all major plant organs—leaves, stems, roots, and reproductive parts. These include widely used traits such as specific leaf area and plant height, but also traits that are rarely reported, especially below‐ground traits related to root morphology, as well as mechanical properties, phenology, and microbial associations. In addition to raw measurements, species are annotated with categorical descriptors (e.g., life form, photosynthetic pathway, and successional status), and species‐level values taken from a Mediterranean flora, for key traits such as reproductive phenology and maximum height. To support analyses that account for environmental variability, each observation is linked to detailed descriptors of the plot where the individual was sampled, including climate data, soil physicochemical properties, and disturbance regime. Full metadata on sampling protocols and measurement methods are provided for every trait and environmental variable. FAIRTraits was built in compliance with the FAIR principles of data management (Findable, Accessible, Interoperable, and Reusable). Metadata are described using the Ecological Metadata Language (EML); trait definitions are standardized using community‐endorsed semantic resources. The data are archived across two interoperable repositories: GBIF (via Darwin Core and trait‐specific extensions) for taxon–trait associations and InDoRES for environmental and contextual data. These efforts ensure long‐term preservation, data traceability, and seamless integration with plant trait databases such as BROT or TRY, and cross‐organism initiatives such as the Open Traits Network or the Encyclopedia of Life. FAIRTraits offers a robust, richly documented, and reusable resource for investigating plant functional strategies, trait–environment relationships, and scaling from individuals to communities and ecosystems. It also provides a concrete example of how trait datasets can meet the highest standards of data quality and interoperability—serving as a model for future community‐led initiatives in functional ecology. The FAIRTraits database is released under the CC‐BY Attribution 4.0 International license.

## CONFLICT OF INTEREST STATEMENT

The authors declare no conflicts of interest.

## Supporting information


Data S1:

## Data Availability

Metadata and data are available in Data S1 as Supporting Information for this paper. In addition, metadata and data are archived in the GBIF (https://www.gbif.org/fr/) and InDoRES (https://www.indores.fr/) repositories as described in section Class V.D.1. of Metadata S1. Code is available in Zenodo at https://doi.org/10.5281/zenodo.17073323.

