# Supplementary material for: FAIRTraits: An enriched, FAIR‐compliant database of plant traits from Mediterranean populations of 240 species
Source: Ecology. 2025 Sep 24;106(9):e70219. doi: 10.1002/ecy.70219 (PMC12460199; doi:10.1002/ecy.70219)
Supplement: Supplementary file 1 — Data S1: [file ECY-106-e70219-s001.zip › MetadataS1.pdf]

Data Paper published in *Ecology*, an Ecological Society of America journal

Metadata S1

FAIRTraits: An enriched, FAIR-compliant database of plant traits from Mediterranean populations of 240 species

Éric Garnier, Léo Delalandre, Jules Segrestin, Karim Barkaoui, Elena Kazakou, Marie-Laure Navas, Denis Vile, Cyrille Violle, Maud Bernard-Verdier, Marine Birouste, Alain Blanchard, Iris Bumb, Pablo Cruz, Sandrine Debain, Adeline Fayolle, Claire Fortunel, Karl Grigulis, Gérard Laurent, Sandra Lavorel, Francisco Lloret, Ignacio M. Pérez-Ramos, Iván Prieto, Catherine Roumet

Class I. Data Set Descriptors

A. Data set identity

FAIRTraits: an environmentally, methodologically and semantically enriched database of plant traits from 1,955 Mediterranean populations of 240 species belonging to 155 genera and 48 families.

B. Data set identification code

FAIRTraits was elaborated as a collection of 10 basic elements corresponding to the various aspects of the database (Table 1). The final format under which the data are published is described in Table 9 (section Class V.D.1.).

Table 1. Basic elements of the FAIRTraits database, with their type (data or metadata), the repository in which the corresponding data are stored, and a short description of their contents and state. See further tables below for details on each element.

Basic element	Type	Repository of destination	Contents
**FAIRTraits_Authors.txt**	Metadata	InDoRES, GBIF	information on contributors to the database
**FAIRTraits_Projects.txt**	Metadata	InDoRES, GBIF	information on projects during which data were collected
**FAIRTraits_Taxon.txt**	Data	GBIF ("Checklist" Darwin Core archive)	information on taxa found in the database with selected characteristics
**FAIRTraits_TraitValues.txt**	Data	GBIF ("Occurrence" Darwin Core archive)	the core file with trait values and relevant information (semantics, methods, etc.)
**FAIRTraits_MeteoSitesMonth.txt**	Data	InDoRES	data on monthly averages of meteorological variables from the meteorological stations closest to the six sites
**FAIRTraits_MeteoSitesYear.txt**	Data	InDoRES	data on yearly averages of meteorological variables from the meteorological stations closest to the six sites
**FAIRTraits_LocalEnvironment.txt**	Data	InDoRES	information on type of soils, treatments, disturbance and soil properties of plots in which environmental data were collected
**FAIRTraits_PlotCorrespondence.txt**	Data	InDoRES	correspondence between plots in which traits were measured and plots in which soil data were collected
**FAIRTraits_DescripEnvVariables.txt**	Metadata	InDoRES	a description of variables, methods and items found in files FAIRTraits_MeteoSitesMonth.txt, FAIRTraits_MeteoSitesYear.txt; FAIRTraits_LocalEnvironment.txt
**FAIRTraits_PublishedPapers.txt**	Metadata	InDoRES	a list of scientific articles in which subsets of the database have been used

C. Data set description

1. Originators

Éric Garnier, Marie-Laure Navas, Elena Kazakou, Cyrille Violle and Catherine Roumet.

2. Abstract

Trait-based ecology relies on high-quality, well-documented data to explore how plant traits relate to environmental conditions, community assembly, and ecosystem functioning. However, the reuse and synthesis of trait data across studies remain limited by several constraints: a lack of detailed metadata, heterogeneous protocols, absence of individual-level measurements, and underrepresentation of certain trait types - particularly below-ground traits. Many existing datasets also lack the environmental details necessary to investigate trait-environment relationships at local scales. Here, we present FAIRTraits, a comprehensive dataset that addresses these limitations by compiling 189,452 records of quantitative trait measurements collected between 1997 and 2023 from 1,955 populations of 240 vascular plant species in the Northern Mediterranean Basin, a region known both for its exceptional biodiversity and as a climate change hotspot. All data were collected by a single research group using consistent and well-documented field and laboratory protocols, ensuring internal consistency across traits, species, sites, and years. FAIRTraits includes 180 traits measured at the individual or replicate level, with no aggregation. It features an unprecedented diversity of traits spanning all major plant organs - leaves, stems, roots and reproductive parts. These include widely used traits such as specific leaf area and plant height, but also traits that are rarely reported, especially below-ground traits related to root morphology, as well as mechanical properties, phenology, and microbial associations. In addition to raw measurements, species are annotated with categorical descriptors (e.g., life form, photosynthetic pathway, successional status), and species-level values taken from a Mediterranean flora, for key traits such as reproductive phenology and maximum height. To support analyses that account for environmental variability, each observation is linked to detailed descriptors of the plot where the individual was sampled, including climate data, soil physico-chemical properties, and disturbance regime. Full metadata on sampling protocols and measurement methods are provided for every trait and environmental variable. FAIRTraits was built in compliance with the FAIR principles of data management (Findable, Accessible, Interoperable, and Reusable). Metadata are described using the Ecological Metadata Language

(EML); trait definitions are standardized using community endorsed semantic resources. The data are archived across two interoperable repositories: GBIF (via Darwin Core and trait-specific extensions) for taxon–trait associations and InDoRES for environmental and contextual data. These efforts ensure long-term preservation, data traceability, and seamless integration with plant trait databases such as BROT or TRY, and cross-organism initiatives such as the Open Traits Network or the Encyclopedia of Life. FAIRTraits offers a robust, richly documented, and reusable resource for investigating plant functional strategies, trait–environment relationships, and scaling from individuals to communities and ecosystems. It also provides a concrete example of how trait datasets can meet the highest standards of data quality and interoperability—serving as a model for future community-led initiatives in functional ecology. The FAIRTraits database is released under the CC-BY Attribution 4.0 International license.

D. Key words/phrases: biodiversity standards; ecosystem properties; environmental conditions; FAIR principles; Mediterranean Biogeographic Region; leaf, stem, root and reproductive traits; plant functional traits; gas exchange; litter mass loss; phenology; terminological resources; trait-based ecology

Class II. Research origin descriptors

A. Overall project description

1. Identity

FAIRTraits compiles and standardizes data collected on six sites located at the north-western edge of the Mediterranean Biogeographic Region, in southern France (five sites) and north-eastern Spain (one site), during the course of 12 research projects (see Table 2 in section Class II.A.6.) conducted within the same research group at CEFE.

2. Originators

Éric Garnier conceived and coordinated the project. Éric Garnier, Marie-Laure Navas, Catherine Roumet, Elena Kazakou Sandra Lavorel and Cyrille Violle elaborated the various research projects leading to the collection of data. All co-authors provided data which were first assembled in a preliminary data set by Jules Segrestin, who also conducted the outlier detection procedure

on the finalized database. Éric Garnier finalized the compilation of data in raw data files, Léo Delalandre elaborated the scripts to format the database in its final form, and Karim Barkaoui conducted the analyses describing the content of the database and prepared the corresponding illustrations.

3. Period of study

The data were collected between 1997 to 2023, during different discrete measurement campaigns at each site.

4. Objectives

To provide a database of plant traits at the individual replicate level, for a broad range of traits collected on individuals from 1,955 Mediterranean populations of 240 species. Methodological details (sampling protocols and measurement methods) are provided for each record, and the interpretation of trait values will be enhanced by the availability of information on the climatic conditions of the sites, soil properties and on disturbance regimes of the plots in which the sampled individuals were collected. A short summary of the rationale underlying the projects during which data were collected is given in section Class II.A.6. (Table 2 and following text; see also the FAIRTraits_Projects.txt file).

The FAIR (Findable, Accessible, Interoperable and Reusable) guiding principles were followed to ensure maximum visibility and reusability of these data.

5. Abstract

FAIRTraits was conceived as a “Grassroots initiative”, i.e. an initiative led by a cohesive group of scientists focused on specific goals (Aubin et al. 2020). This approach to data management plays a crucial role in making high quality datasets, by a thorough account of the heterogeneity and complexity of ecological data at a scale which remains manageable (Aubin et al. 2020). In FAIRTraits, this has resulted in special efforts focused on three main aspects, to give users: (1) information on the environmental factors (climate, disturbance, soil) controlling trait values at the local scale at which the traits were measured, (2) information on sampling protocols and methods at the level of *each* attribute, while, when given, this information is generally available at the level of the whole data set (e.g. in the Ecological Metadata Language: cf. Michener et al. 1997),

and (3) accurate definitions of all variables in the database using relevant terminological resources; when definitions were missing, these were coined and explicitly attached to the corresponding variable.

We also wanted to adopt a fully open science approach (Hampton et al. 2015), so that FAIRTraits could be redistributed without restrictions. We chose to store the core data of FAIRTraits (the two files with information on traits and taxa) in the GBIF repository (https://www.gbif.org/), one of the largest repositories for biodiversity data, using Darwin Core basic elements (Wieczorek et al. 2012) and extensions specifically devoted to traits. Environmental data which could not be mapped to Darwin Core are stored in the InDoRES InEE-CNRS institutional repository (https://www.indores.fr/), with proper cross references to the GBIF Darwin Core archives. FAIRTraits data can thus be easily harvested by domain specific databases, such as BROT (Tavşanoğlu and Pausas 2018) or TRY (Kattge et al. 2020), which are respectively regional (Mediterranean) and global scale bases of plant traits, or by cross-organism initiatives such as the Encyclopedia of Life (Parr et al. 2014) or the Open Traits Network (Gallagher et al. 2020). Harvesting data sets from recognized repositories is a guarantee that these are identified with an unequivocal identifier (e.g. a doi or a set of dois), which ensures data traceability including easy identification of their origin. This can be particularly useful and save a substantial amount of time during the process of provenance tracking and attribution of credit in the context of wide ranging compilations of data (cf. Poisot et al. 2019).

6. Sources of funding

Data were collected during the course of 12 projects (Table 2), whose specific objectives are briefly described below.

Table 2. Research projects during which data were collected, with their principal investigator, the period and source of funding. Details on the specific objectives of each project can be found in the text below and in the FAIRTraits_Projects.txt file (see Table 1).

Project acronym	Full name of project	Principal investigator	Period of study	Source of funding
**MELODY**	Mediterranean Landscapes in a Changing World: Coupling Dynamics and Functional Analyses	Serge Rambal	1997-2003	ICSU – IGBP – Core Research Project
**DynEcoMed**	Dynamique des Ecosystèmes Méditerranéens dans un Monde Changeant	Sandra Lavorel	2001-2006	CNRS – Laboratoire Européen Associé CEFE – CREAF
**INDIGO**	Une Nouvelle Perspective sur les Indicateurs de Diversité Végétale : Application à l'Etude des Conséquences de la Déprise Agricole et des Valeurs d'Usage des Prairies	Eric Garnier	2002-2006	MATE – Programme « Action Publique, Agriculture et Biodiversité »
**VISTA**	Vulnerability of Ecosystem Services to Land Use Change in Traditional Agricultural Landscapes	Sandra Lavorel	2002-2006	European Union – Contract n°EVK2-2001-000356

Project acronym	Full name of project	Principal investigator	Period of study	Source of funding
**GEOTRAITS**	Impacts des Traits Fonctionnels des Espèces Végétales sur les Cycles Biogéochimiques	Eric Garnier	2004-2007	ACI – FNS Ecosphère Continentale (PNBC)
**DivHerbe**	Structure, Diversité et Fonctionnement : des Clefs Multi-Echelles pour la Gestion des Prairies Permanentes	Eric Garnier	2006-2009	INRA – AIP EcoGer
**RESPIRS**	The Root Economics Spectrum and InfraRed Spectroscopy	Catherine Roumet	2010-2012	FRB – Programme « Petits et percutants »
**O2LA**	Organismes et Organisations Localement Adaptés	Marie-Laure Navas	2010-2015	ANR – Contract n°09-STRA-09
**CASCADE**	Interactions Trophiques et Fonctionnement des Ecosystèmes Terrestres : une Approche Fonctionnelle et Chimique	Eléna Kazakou	2013-2014	CNRS – INSU – InEE – INC – Initiative structurante EC2CO
**PhenBiom**	Phenology and Biomechanics of Plants in the Mediterranean	Eric Garnier	2013-2018	CNRS – Core support funding
**COMODO**	Connecter les Mondes Verts et Bruns : Ecologie Trophique des Détritivores et des Phytophages en Prairie Permanente	Sylvain Coq	2015-2017	CNRS – INSU – Initiative structurante EC2CO
**FageAnnuals**	Functional Ecology of Annual Plant Species from a Mediterranean Rangeland	Cyrille Violle	2021-2023	European Union - ERCStG-2014-639706

MELODY was a unifying program focusing on biodiversity dynamics at the regional scale of the French Mediterranean area.

DynEcoMed was aimed at understanding and predicting the dynamics of vegetation structure and functioning in Mediterranean ecosystems undergoing land use and climate changes.

INDIGO aimed to (i) analyze the consequences of the decrease in land use by agriculture in some areas on biological diversity; (ii) relate these changes in diversity to the functioning of ecosystems and services delivered when relevant; and (iii) propose a new methodology to assess the effects of changes in agricultural practices on vegetation.

VISTA aimed to produce an integrated assessment of the vulnerability of European traditional agro-pastoral landscapes to land use and climate change to assist land managers and regional policy makers towards sustainable development.

GEOTRAITS objectives were to analyze the impacts of differences in plant functional characteristics as assessed through the values of their traits, on biogeochemical cycles.

DivHerbe objectives were to understand the mechanisms that control species diversity and structural heterogeneity in plant communities, and propose simple indicators aimed at managing grassland diversity according to combined environmental and production objectives.

RESPIRS objectives were to test on a large number of temperate, Mediterranean and tropical species: i) the existence of a root economics spectrum consisting of key chemical and structural traits enabling the prediction of root respiration and decomposition, and ii) the potential of near infrared spectroscopy to characterize root diversity.

O2LA was an interdisciplinary research project aiming to characterize the added value of diversification (of biological resources, management practices, knowledge transfers) in French livestock production systems.

CASCADE was designed to understand the response of the soil-plant system to herbivory and identify the consequences of plant responses on the quality of the resources for herbivores on the one hand, and on decomposers through the litter produced on the other hand.

PhenBiom was a research project aiming at integrating plant reproductive phenology and leaf mechanical defense into the phenotypic space of Mediterranean plants.

COMODO objectives were to describe, understand and predict the structure of trophic networks through the identification of relevant trophic interaction traits. Two coupled ecological networks were studied: plants-herbivores and litter-decomposers.

FageAnnuals was aimed at testing whether species differing in their life history display contrasted trait-environment relationships using the case of annual and perennial species, which often co-occur in communities, but whose life cycles fundamentally differ.

B. Specific subproject description

1. Site description

Data were collected in six sites located at the north-western edge of the Mediterranean Biogeographic Region (Table 3; Figure 1): five in southern France (Cazarils, Hautes Garrigues du Montpelliérais, La Fage, Les Agros and Camp Redon), and one in north-eastern Spain (Garraf). All sites are located *in natura* except Camp Redon, which is an experimental garden. Selected characteristics of these sites are given in Table 3, and views of representative areas are shown in Figure 1.

Table 3. Selected characteristics of the six study sites where data have been collected. Temperature and precipitation data were taken from the meteorological station closest to each site. Soil type follows the WRB classification (World Reference Base for Soil Resources 2022). More details can be found in the FAIRTraits_LocalEnvironment.txt, FAIRTraits_MeteoSitesMonth.txt and FAIRTraits_MeteoSitesYear.txt files.

Characteristics	Cazarils	Hautes Garrigues du Montpelliérais	La Fage	Les Agros	Garraf	Camp Redon
**Country, Region**	France, Bas Languedoc	France, Bas Languedoc	France, Grands Causses	France, Provence	Spain, Catalonia	France, Bas Languedoc
**Latitude (decimal degree)**	43.764	43.852	43.927	43.625	41.304	43.638
**Longitude (decimal degree)**	3.703	3.925	3.099	6.586	1.892	3.862
**Altitude range (m a.s.l.)**	240-310	100-160	765-830	405-465	250-350	52
**Approximate site size (m x m)**	1600 x 800	4000 x 4000	1900 x 1600	600 x 300	2500 x 2500	40 x 40
**Distance of meteorological station from study site (km)**	3.3	14.7	1.0	1.5	15.0	0.03
**Mean annual temperature (°C)**	13.5	13.5	10.0	13.6	16.1	14.6
**Mean annual precipitation (mm)**	1075	1014	1024	1041	588	780
**Soil type**	Leptosol	Calcaric Cambisol	Dolomitic Rendzic Leptosol and Rendzic Leptosol	Calcaric Cambisol	Calcisol (Hypocalcic)	Calcaric Cambisol
**Average soil pH**	7.1	8.3	7.5	8.0	8.2	7.8
**Reference for detailed description of site**	Le Floc'h et al. 1998	Garnier et al. 2004	Molénat et al. 2005	Garnier et al. 2001	Lloret and Vilá 2003	Barkaoui et al. 2016, Kazakou et al. 2007

At all sites, the climate is classified as Mediterranean, with mild winters and (relatively) dry summers: it spans from "dry sub-humid" in Garraf, to "humid" in Cazarils, Hautes Garrigues, Les Agros, and Camp Redon, and while still classified as Mediterranean, the climate in La Fage is at the limit of the temperate zone (cf. Figure 1; see Daget 1977 for bioclimate categories). In the six sites, the bedrock is limestone and soils show neutral to slightly basic pH (Table 3). Sites have undergone a strong and long-lasting human imprint (see short description below). The vegetation is generally engaged in highly dynamic processes resulting from changes in land use (e.g. abandonment of cultivation, grazing, fertilization), resulting in a complex mosaic of herbaceous and woody components (Figure 1).

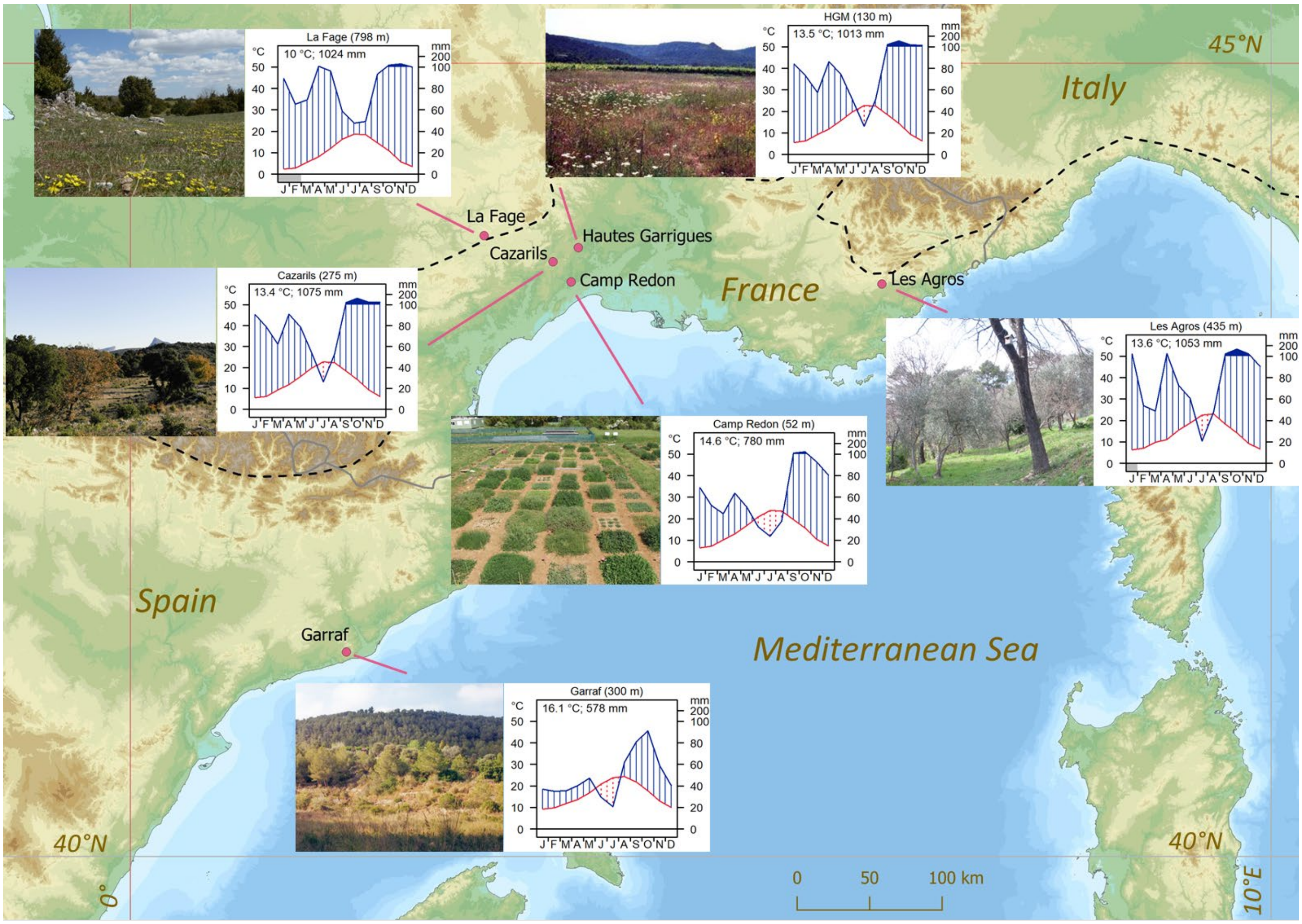

Figure 1. Map showing the location of the six sites where data have been collected. Views of representative areas of the six sites with the corresponding climate diagrams (cf. Walter et al. 1975; blue line: rainfall, red line: temperature) are added. On these diagrams, dry periods correspond to the area with red dots (monthly rainfall <2 × average monthly temperature: cf. Walter et al., 1975). At the bottom of each plot, a grey bar represents months with mean minimum temperatures below 0°C. Table 3 for further details. Colors on the background map correspond to altitudinal variations from low (green) to high (brown) altitudes. The black dashed line shows the northern limit of the Mediterranean Biogeographic Region (cf. Tison et al. 2014). Photo credits for each site photo: La Fage (upper left) by Maud Bernard-Verdier; Les Agros (middle right by Joël Garnier; Hautes Garrigues, Cazarils, Camp Redon, and Garraf by Eric Garnier.

A short description of each site is given below.

Cazarils, a part of the "Domaine de Roussières", consists of a large clearing in an otherwise mostly wooded area, with *Quercus pubescens* and *Q. ilex* as dominant tree species. This clearing, which was cultivated until the mid-20th century, is surrounded by degraded Mediterranean shrublands ("garrigue") that were regularly and historically cut for fuel wood collection. Over the past 80 years or so, recreational hunting, selective clearcutting and burning, and extensive

grazing by sheep and goats have been the main disturbances at this site. Former and current land uses have resulted in a complex spatial organization of the vegetation with plant communities composed of species from different growth and life forms (Le Floc'h et al. 1998, Navas et al. 2010).

Hautes Garrigues du Montpelliérais consists of a collection of twelve old-fields located in a matrix of cultivated vineyards, other less represented crops and woodlands. These fields were all previously vineyards, which, following removal of the vines, were abandoned 2 to 42 years prior to 2000. The vegetation shifts from predominantly annual species in recently abandoned fields (less than 5 years) to herbaceous perennials in fields of intermediate age (6-15 years), and to a mixture of herbaceous and woody perennials in fields older than 25 years (see Garnier et al. 2004 for details).

La Fage is an experimental farm managed by the French National Research Institute for Agriculture, Food and the Environment (INRAE). The area was probably cultivated until the 19th century, at least partially. It was then extensively grazed until 1978, both by local and transhumant sheep herds, allowing woody species to recolonize a large part of the area. Since 1978, it has been subdivided into paddocks either grazed by sheep at a low stocking rate, or fertilized and grazed by sheep at a high stocking rate, or fenced to prevent grazing. The vegetation consists primarily in rangelands dominated by perennial grasses and forbs, with a small woody component covering approximately 20–25% of the area (see Molénat et al. 2005 for details).

Les Agros consists of a series of south facing terraces located on the southern edge of a large limestone plateau, which were formerly cultivated mainly with olive trees until the second half of the 19th century. Until approximately 40 years ago, the terraces were occasionally grazed by sheep. Since then, they have been regularly cleared. The vegetation is mostly herbaceous with numerous shrubs and olive tree resprouts, surrounded by a mixed *Pinus halepensis – Quercus ilex* woodland (see Garnier et al. 2001 for details).

Garraf (Garraf Natural Park) is a karstic massif where the vegetation is a mosaic of shrublands (including "garrigue") and *Pinus halepensis* open forests growing on rocky soils and on terraces that were established over marls and cultivated in the past centuries. Agricultural abandonment started at the end of the 19th century and was completed around the mid-20th century. Several

wildfires have occurred since then, particularly in 1982 and 1994, affecting most of the area. Shrubs (macrophanerophytes), scrubs (nanophanerophytes) and herbaceous species are important components of the community (see Lloret and Vilá 2003 for details).

Camp Redon is a 2.5 ha experimental garden attached to the CEFE research unit, located in the outskirts of the Montpellier city. Data for FAIRTraits are derived from two experiments conducted on small areas of this garden. Plants were cultivated in plots previously weeded and in which the 5cm upper layer of soil had been removed. The first experiment ("PDM") was conducted in 2004 and 2005 on 18 species from the old-field succession of the Hautes Garrigues du Montpelliérais site described above, grown under high and low nitrogen supply (Kazakou et al. 2007 for details). The second one ("O2LA") was conducted in 2012 and 2013 on a selection of perennial species from the La Fage Mediterranean rangeland described above, grown at two levels of water supply (Barkaoui et al. 2016; Fort et al. 2017 for details).

2. Experimental or sampling design

a. Design characteristics

Spatial design: in the case of the five *in natura* sites, delimited topographic zones of various areas named "plots" were recognized, which are the spatial units in which individuals were selected for trait measurement ("traitPlots" hereafter; 106 traitPlots across all five sites). Each plot was assigned to a single modality of a specific treatment (taken in a broad sense), which differ in each site. In the case of Camp Redon, there were 144 traitPlots (1.20 x 1.20 m) overall (72 at each level of nitrogen supply) in the PDM experiment, and 76 traitPlots (1 x 1.20 m) overall (64 and 12 in the "ambient" and "low" water supplies, respectively) in the O2LA experiment. Each trait record in the database is attached to one and only one of these traitPlots.

Species selection for trait measurements: in the case of the five *in natura* sites, measurements were conducted on the most abundant species from each site (Figure 2a). When the objective was to test the effects of community functional structure on ecosystem properties, we sampled species that collectively made up at least 80% of vegetation cover and/or biomass in each treatment, when relevant (see Garnier et al. 2004, Pakeman & Quested 2007). In the case of the two experiments conducted in Camp Redon, species were selected based on their abundance in the different stages of the chrono sequence studied in Hautes Garrigues du Montpelliérais (PDM), and on their abundance in the low stocking rate, unfertilized treatment in La Fage (O2LA). Figure

2a shows the number of "populations" on each site, defined as the combination of species x site x plot x treatment x "field campaign" (which can cover a period from one day to a few weeks

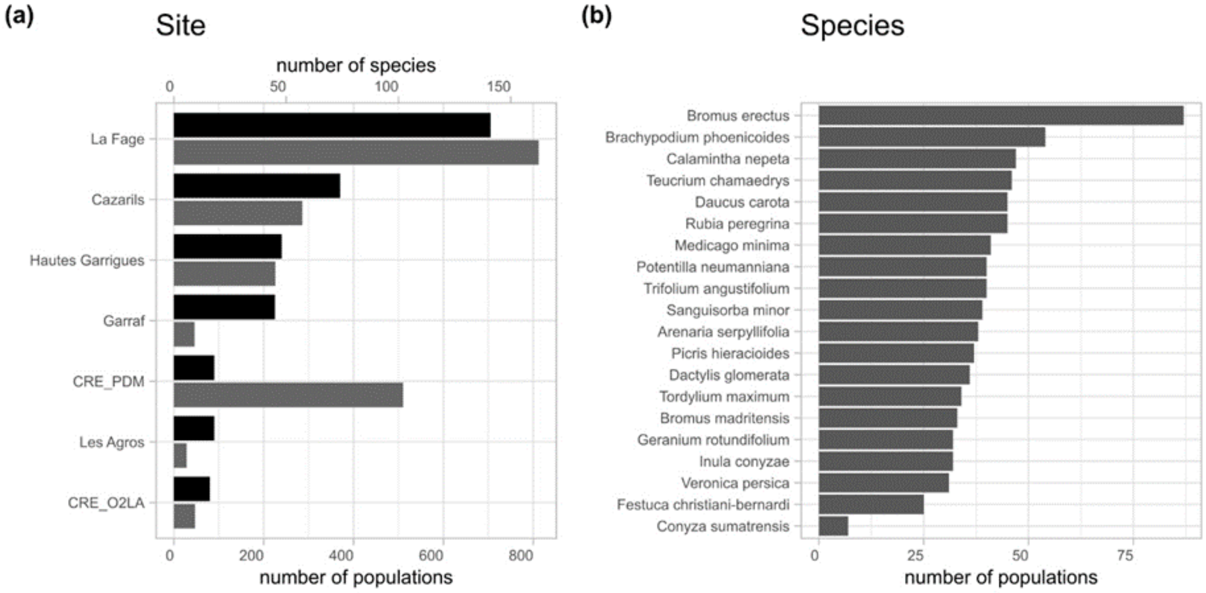

during a given year).

Figure 2. (a) cumulated number of species (black bars, upper x-scale) and populations (grey bars lower x-scale), grouped by site, and (b) cumulated number of populations for the 20 most represented species in the FAIRTraits database.

Traits were measured on 171 herbaceous (71 therophytes, 86 hemicryptophytes and 14 geophytes) and 69 woody (35 chamaephytes, 11 deciduous and 18 evergreen phanerophytes, and 5 lianas) species (detailed information on taxa given in the FAIRTraits_Taxon.txt file: cf. Table 4 in section Class IV.B.1. below). Asteraceae (36 species), Poaceae (34 species), Fabaceae (29 species), Lamiaceae (16 species) and Rosaceae (14 species) are the five most represented botanical families in the database. With one exception (Rosaceae), this corresponds to the representation of families in BROT 2.0, a database of plant functional traits across the Mediterranean Basin (Tavşanoğlu and Pausas 2018). The five species with the highest number of populations are *Bromus erectus* (Poaceae, 87 populations), *Brachypodium phoenicoides* (Poaceae, 54 populations), *Calamintha nepeta* (Lamiaceae, 47 populations), *Teucrium chamaedris* (Lamiaceae, 46 populations) and *Daucus carota* (Apiaceae, 45 populations) (Figure 2b).

Environmental factors: environmental data (detailed below in Table 7, section Class IV.B.4.) are provided at the site and/or at the plot level. At the site level, 30-year average values of a number

of monthly and yearly climatic variables were compiled from meteorological stations closest to the sites. Within each site, the local environment was more precisely characterized in two ways. First, the soil type (cf. Table 3 in section Class II.B.1.), disturbance regime (type, frequency and intensity) and a qualitative characterization of resource supply (nutrient or water) are given at the treatment level (i.e. the same values of the different variables were assigned to all plots attached to this treatment). Second, we selected a subset of 84 plots ("soilPlots" hereafter) representative of either a specific treatment or a particular topographic zone, in which 11 quantitative soil variables were quantified (listed in Table 7 in section Class IV.B.4.). Each traitPlot in which traits were assessed was then associated with one and only one of these soilPlots.

b. Data collection period

Across the whole database, data collection took place between 1997 and 2023. The main sampling periods differ among sites, in relation with the sites selected for the various projects listed in Table 2 (section Class II.A.6.): 1997-2001 in Cazarils with some additional sampling in 2017 and 2023; 1998-1999 in Les Agros; 2000-2004 in Hautes Garrigues du Montpelliérais; 2001 in Garraf; 2006-2022 in La Fage; 2004-2006 (PDM) and 2012-2013 (O2LA) in Camp Redon. Samples of plant vegetative organs were mostly collected between March and July, corresponding to the main growing season under the Mediterranean climate prevailing at the different sites. Seed production and seed traits were assessed when seeds were ripe, while plant litter was collected at peak litter production, in the summer and early autumn. The exact date at which measurements were taken is provided for 97% of the data in the FAIRTraits_TraitValues.txt file.

3. Research methods

For each record in the FAIRTraits_TraitValues.txt file database, a short description of the sampling protocol (field "samplingProtocol") and of the method used to determine the trait value with relevant references (field "measurementMethod") are given (see Table 5 in section Class IV.B.1. for the structure of this file). This was also done for the environmental data in the three corresponding files (see Table 7 in section Class IV.B.4.). Given the large number of traits measured (180 overall: section Class IV.B.2.), we give only a short overview of methods in this section, and direct the reader to the relevant data files for further details.

a. Field/laboratory

Traits: for above-ground vegetative and reproductive traits, sampling and measurement methods generally followed Cornelissen et al. (2003), a handbook completed with the participation of some of the contributors to the present database, during the period of the first research projects which led to the collection of data synthesized in FAIRTraits. For below-ground traits, we mostly followed the "starting guide to root ecology" (Freschet et al. 2021), which assembles methods developed and improved over the last two decades or so, and to which several contributors to FAIRTraits have participated. Traits were mostly determined on healthy individuals of the targeted species found in full light conditions. Some traits were measured non-destructively on-site on intact, undisturbed individuals (plant height, lateral spread, reproductive phenology, photosynthetic rate, etc.), while for traits requiring further processing in the laboratory (leaf area and mass, root length and mass, seed shape and mass, leaf and root nutrient content, etc.), as far as possible, material free from herbivore or pathogen damage was collected. The number of samples (individuals or parts thereof) on which traits were determined varied according to the trait (cf. Table 3 in Cornelissen et al. 2003 for above-ground traits). The exact number of replicates used for each trait can be retrieved from the FAIRTraits files, in which the finest level of replication is conserved.

Environmental data: at the site level, climate was described using data from the meteorological station closest to the site (i.e. one value *per site* for each variable retained). Disturbance type, frequency and intensity were assessed at the treatment level following Garnier et al. (2007), and soil samples from different layers (specified in the FAIRTraits_LocalEnvironment.txt) were collected in the 84 soilPlots described above (section Class II.B.2.a.). These samples were sent to the INRAE "Laboratoire d'Analyses des Sols d'Arras" (https://las.hautsdefrance.hub.inrae.fr/) for analyses. A short summary of methods is given in the FAIRTraits_LocalEnvironment.txt file for each variable, and a full description can be found in the "catalogue analytique" of the INRAE laboratory available at: https://las.hautsdefrance.hub.inrae.fr/prestations/catalogue-analytique.

b. Instrumentation

A whole range of tools has been used to determine the 180 traits of the database: human eyes (phenological records), rulers and tape measures (plant height, lateral spread, etc.), precision balances (masses), flatbed scanners coupled with image analysis systems (leaf area, root length and diameter, etc.), portable gas exchange systems (photosynthesis, root respiration), elemental

(carbon, nitrogen content) and colorimetric analyzers (phosphorus content), mass spectrometers (element isotopic fractions), etc.. In addition, some trait values were derived from calculations based on raw data (rooting depth, litter mass remaining standardized after a given period, etc.). The brand and model of the devices used as well as the calculations procedures when relevant, are given for each record in the “measurementMethod” field of the database (see Table 5 in section Class IV.B.1.).

c. Taxonomy and systematics

See section Class IV.B.3. below for taxonomy and additional features retained to describe taxa.

d. Permit history

Cazarils: a formal permit was issued by the Hérault Departmental Council, the administrative entity that owns and manages the “Domaine de Roussières”, to conduct field work and collect samples.

Hautes Garrigues du Montpelliérais: the owners of the land where the twelve old-fields were located were contacted and gave verbal permission to conduct field work and collect samples on their properties until the INDIGO and VISTA projects came to an end.

Les Agros: the owner gave a verbal permission to conduct field work and collect samples on this private property. He participated in the collection of leaf life span data at this site, and was offered co-authorship on the related publication.

Garraf: a formal permit was issued by the administration of the Garraf National Park to CREAF to conduct field work and collect samples.

La Fage: the “Génétique Animale” department of INRAE, who rules the experimental farm, has given a long-term agreement to CEFE researchers to conduct research in this site. The staff of the farm is regularly associated as co-authors to publications resulting from the work conducted there.

Camp Redon: as an entity attached to the CEFE laboratory, permission to use specific areas of the experimental garden is discussed with the technical staff in charge of its management when required.

e. Legal/organizational requirements: all intermediate and final reports of projects listed in Table 2 (section Class II.A.6.) were delivered as required.

4. **Project personnel**

Co-authors of this data paper, laboratory technicians and undergraduate students from the CEFE and AGIR research units.

Class III. Data set status and accessibility

A. Status

1. **Latest update:** 19 March 2025
2. **Latest archive date:** Data are archived as described in section Class V.D.1.
3. **Metadata status:** last update on 19 March 2025
4. **Data verification:** see section Class V.B.

B. Accessibility

1. **Storage location and medium**

The different files constituting the database are available as Supporting Information with this *Ecology* data paper. Metadata are available as EML, XML and rtf files on the GBIF portal (https://www.gbif.org/fr/), and archived in the InDoRES metadata catalog (https://cat.indores.fr/geonetwork/srv/eng/catalog.search#/home). Data are archived in two repositories: GBIF for taxa and trait data formatted using the Darwin Core data standard (Table 9 in section Class V.D.1. for details), and InDoRES (https://www.indores.fr/) for environmental data. Code (Delalandre 2025) is available in Zenodo at https://doi.org/10.5281/zenodo.17073323 (see Table 8 in section Class V.C. for details).

2. **Contact persons**

a. *Eric Garnier:*

CEFE, Univ Montpellier, CNRS, EPHE, IRD, Montpellier, France

Phone: (+) 33 4 67 61 32 42

Email: eric.garnier@cefe.cnrs.fr

b. *Cyrille Violle:*

CEFE, Univ Montpellier, CNRS, EPHE, IRD, Montpellier, France

Phone: (+) 33 4 67 61 33 42

Email: cyrille.violle@cefe.cnrs.fr

c. *Karim Barkaoui:*

CIRAD, UMR AMAP, Montpellier, France

Phone: (+) 33 6 75 59 82 40

Email: karim.barkaoui@cirad.fr

d. *Christelle Dantec:*

CEFE, Univ Montpellier, CNRS, EPHE, IRD, Montpellier, France

Phone: (+) 33 4 67 61 32 56

Email: chrsistelle.dantec@cefe.cnrs.fr

3. Copyright restrictions

The FAIRTraits database is released under the CC-BY Attribution 4.0 International license.

4. **Proprietary restrictions**

We request that users of the FAIRTraits database or portion thereof to cite this data paper. In addition, when relevant, users may want to cite the original study in which the data have been collected and presented in the first place. A list of papers with associated projects (Table 2 in section Class II.A.6.) is given below (section Class V.E.) and is available in the FAIRTraits_PublishedPapers.txt file.

5. **Costs:** There are no costs associated with using the FAIRTraits database.

Class IV. Data structural descriptors

A. Data set files

1. Identity

Following the approach presented by Schneider et al. (2019), data have been divided into six files (see Table 1 in section Class I.B.). A core file (FAIRTraits_TraitValues.txt) containing trait values and contextual information, with five additional files giving detailed information on taxa (FAIRTraits_Taxon.txt), climatic variables at the site level (FAIRTraits_MeteoSitesYear.txt for average yearly data and FAIRTraits_MeteoSitesMonth.txt for average monthly data), local environmental conditions (FAIRTraits_LocalEnvironment.txt), and the correspondence between plots in which traits and soil variables were measured (FAIRTraits_PlotCorrespondence.txt: see section Class II.B.2.a.). Additional "metadata files" (listed in Table 1, section Class I.B.) give

detailed descriptions of the fields that compose the three files describing the site and local environments (FAIRTraits_DescripEnvVariables.txt), and a file listing the published articles resulting from the use of subsets of the database is also provided (FAIRTraits_PublishedPapers.txt; cf. section Class.V.E.). Note that the information described in the metadata files listing the authors and the projects will be completed directly in the EML fields of the GBIF and InDoRES repositories. The sizes of these files are thus not given in the next section.

2. Size of data files

***FAIRTraits_TraitValues.txt*:** 189,452 rows (excluding the header); 34 columns; 129 Mo. In this core file, the data are in long format where each row represents values and basic metadata associated with a single trait observation. More detailed metadata are available as extensions to this master file, as recommended in the ecological trait-data Standard (Schneider et al. 2019).

***FAIRTraits_Taxon.txt*:** 240 rows (excluding the header); 18 columns; 72 Ko.

***FAIRTraits_MeteoSitesMonth.txt*:** 84 rows (excluding the header); 18 columns; 10 Ko.

***FAIRTraits_MeteoSitesYear.txt*:** 7 rows (excluding the header); 17 columns; 2 Ko.

***FAIRTraits_LocalEnvironment.txt*:** 84 rows (excluding the header); 28 columns; 25 Ko.

***FAIRTraits_PlotCorrespondence.txt*:** 376 rows (excluding the header); 4 columns; 14 Ko.

***FAIRTraits_DescripEnvVariables.txt*:** 50 rows (excluding the header); 6 columns; 9 Ko.

***FAIRTraits_PublishedPapers.txt*:** 59 rows (excluding the header); 5 columns; 16 Ko.

3. Format and storage mode

All files are provided in tab-separated-value format (txt), encoded with UTF_8.

4. Header information

See variable information in section Class IV.B. below.

5. Special characters/fields

In all files, "NA" denotes missing data, while "none" means that there is no relevant value for the attribute (for example, a simple leaf will have a "none" value for the trait "number of leaflets").

Three identifiers were designed for each record: the first one is the unique identifier of the record (verbatimOccurrenceID; required for Darwin Core archives: cf. Table 4 in section Class IV.B.1.), while the other two have been designed to enable users to associate data from different traits at the replicate (verbatimOccurrenceID_sample) and population levels (verbatimOccurrenceID_population).

B. Variable information

1. Taxa

Field identification of taxa was based on local (mostly de Bolòs et al. 1993, Bernard 2008) or national (Coste 1937) floras. Species names (“acceptedNameUsage” in Darwin Core: Table 6) were homogenized using the reference flora for the French Mediterranean region (Tison et al. 2014), while the scientific name (“scientificName” in Darwin Core: Table 6) was taken from the TaxRef (version 16) referential (https://taxref.mnhn.fr/taxref-web/accueil), the most comprehensive taxonomic resource for France (which also covered the 45 species studied in Catalonia). 224 taxa were identified at the species level, 11 at the subspecies level, and 5 at the genus level. The taxon table (described in Table 4) also gives information on life cycle, life form *sensu* Raunkiær (1934), reproductive phenology and geographic distribution of the taxa (all taken from Tison et al. 2014), and on their photosynthetic pathway (mostly derived from the original $^{13}C/^{12}C$ isotopic fraction values available in the database, with additional information given by Colin Osborne). We also provide an original successional stage indicator value for 66 of the species, based on Braun-Blanquet et al. (1952), Escarré et al. (1983) and Max Debussche (pers. com.). Darwin Core terms and classes resulting from the mapping of the variables included in the FAIRTraits_Taxon.txt file are also given in Table 4, as this file is a Darwin Core “Checklist” archive on the GBIF infrastructure.

Table 4. Structure of the FAIRTraits_Taxon.txt file (Taxon). The Darwin Core terms and classes are given for each variable, when available. This Table is available in the metadata file of the Darwin Core “Checklist” archive of the GBIF infrastructure. Units, literature sources and methods are also available in this file.

Name of variable in Taxon file	Variable definition	Darwin Core term	Darwin Core class
**speciesName**	Name of species taken from a local flora (latin binomial without authority)	acceptedNameUsage	Taxon
**taxonRank**	The taxonomic rank of the most specific name in the Darwin Core field “scientificName”	taxonRank	Taxon
**codeSp**	Identification code of the species (4 first letters of genus name and 4 first letters of species name)	taxonID	Taxon
**kingdom**	The full scientific name of the kingdom in which the taxon is classified	kingdom	Taxon
**family**	The botanical family of the species	family	Taxon
**lifeCycle1**	Primary life cycle of species (annual, monocarpic perennial, perennial)	LifeCycleUnstructured	Plinian Core Simple Extension
**lifeCycle2**	Secondary life cycle of species (annual, bisannual, monocarpic perennial, perennial), if any	NA	NA
**lifeForm1**	Primary life form of species *sensu* Raunkiaer (1934)	LifeFormUnstructured	Plinian Core Simple Extension
**lifeForm2**	Secondary life form of species *sensu* Raunkiaer (1934), if any	NA	NA

Name of variable in Taxon file	Variable definition	Darwin Core term	Darwin Core class
**successionalStage**	Successional Stage Indicator Value	HabitatUnstructured	Plinian Core Simple Extension
**plantHeight**	Minimum plant height - Maximum plant height (cm)	NaturalHistoryUnstructured	Plinian Core Simple Extension
**floweringStart**	Beginning of flowering period (month)	StartTimeInterval	Plinian Core Simple Extension
**floweringEnd**	End of flowering period (month)	EndTimeInterval	Plinian Core Simple Extension
**photosyntheticPathway**	Primary mode of atmospheric carbon dioxide fixation (C_3, C_4, CAM)	TrophicStrategy	Plinian Core Simple Extension
**chorology**	Geographic distribution of the species	DistributionUnstructured	Plinian Core Simple Extension
**scientificName**	Scientific name of the species with authority	scientificName	Taxon
**nameAccordingTo**	Identifiant number of the species in TaxRefv16	nameAccordingTo	Taxon
**scientificNameID**	Uniform Resource Identifier of the species in TaxRefv16	scientificNameID	Taxon

2. The TraitValues core file

Table 5 describes columns of the core file (FAIRTraits_TraitValues.txt), which contains trait data and contextual information about the collection of these data (e.g. location identification and coordinates, treatments, date, methods, projects, etc.), trait semantics (entity, quality [see following section], terminological resources, etc.), and the three identifiers described above. Semantics mostly follow Garnier et al. (2017) for above-ground traits, Freschet et al. (2021) for belowground traits and Ely et al. (2021) for leaf gas exchange. Original definitions were coined when necessary. The two last columns of Table 4 show the Darwin Core terms and classes resulting from the mapping with the original names of variables in the FAIRTraits files, a process required to build the Darwin Core "Occurrence" archive on the GBIF infrastructure.

Table 5. Structure of the core file FAIRTraits_TraitValues.txt (TraitValues). The Darwin Core terms and classes are given for each variable. This Table is available in the metadata file of the Darwin Core "Occurrence" archive of the GBIF infrastructure.

Name of variable in TraitValues file	Variable definition	Darwin Core term	Darwin Core class
**speciesName**	Name of species taken from a local flora (latin binomial without authority)	acceptedNameUsage	Taxon
**codeSp**	Identification code of the species (4 first letters of genus name and 4 first letters of species name)	taxonID	Taxon
**family**	The botanical family of the species	family	Taxon
**lifeForm1**	Primary life form of species *sensu* Raunkiaer (1934)	taxonRemarks	Taxon
**siteName**	Name of site	locality	Location
**traitPlotTreatment**	Experimental treatment applied in the plot, if any	occurrenceRemarks	Occurrence
**year**	Year of data collection	year	Event
**month**	Month of data collection	month	Event

Name of variable in TraitValues file	Variable definition	Darwin Core term	Darwin Core class
**day**	Day of data collection	day	Event
**traitName**	The full name of the trait	measurementTraitName	Trait measurement score (v20140515)
**traitAbbreviation**	Abbreviation used for the trait	measurementTraitID	Trait measurement score (v20140515)
**replicateID**	Identification of the replicate measurement	materialSampleID	MaterialSample
**traitValue**	Value taken by the trait for the specified replicate measurement	measurementValue	Trait measurement score (v20140515)
**traitUnit**	Unit in which the trait value is expressed	measurementUnit	Trait measurement score (v20140515)
**entityQuality**	Formal combination of trait entity and quality (entity_quality)	measurementTraitRemarks	Trait descriptor (v20140515)
**traitCategory**	The category to which the trait is assigned (building on McCormack et al. 2017)	measurementTraitCategory	Trait descriptor (v20140515)
**variableType**	The type of variable: a trait, a condition or a statistic	measurementType	Trait descriptor (v20140515)
**basisOfRecord**	The specific nature of the data record	basisOfRecord	Record-level
**termSource**	Terminological resource in which trait formalism and definition were taken, when available	measurementTraitSource	Trait descriptor (v201405015)
**traitLocalIdentifier**	Identifier of the trait in the terminological resource selected	measurementID	Trait measurement score (v20140515)
**traitResourceIdentifier**	URL or URI of the web resource used for trait formalism and definition, or definition when not available	measurementTraitIdentifier	Trait measurement score (v20140515)
**samplingProtocol**	Short description of number of, and conditions in which, samples were taken for a specific measurement	samplingProtocol	Event
**measurementMethod**	Short description of, or reference to, the method used to determine the measurement or characteristics	measurementMethod	Trait measurement score (v20140515)
**experimentalBlock**	A set of experimental units grouped on the basis of their similarity for one or more variables	locationRemarks	Location
**traitPlotID**	Identification code of the plot where trait(s) were determined	locationID	Location
**countryCode**	Standard 2-letter code of the country in which the sites and plots are located (ISO 3166-1-alpha-2)	countryCode	Location
**traitPlotDecimalLatitude**	Latitude of the plot (decimal degree)	decimalLatitude	Location
**traitPlotDecimalLongitude**	Longitude of the plot (decimal degree)	decimalLongitude	Location
**traitPlotAltitude**	Altitude of the plot (meters above sea level)	verbatimElevation	Location
**nameOfProject**	Name of project during which data were collected (cf. Table 2)	eventRemarks	Event
**measurementDeterminedBy**	Name of the contact person responsible for the acquisition of the data	recordedBy	Occurrence
**occurrenceID**	Unique identifier of the data record	occurrenceID	Occurrence
**replicateSampleID**	Identifier of the replicate on which a specific data record was taken	organismID	Organism
**populationID**	Identifier of the population (species x site x plot x treatment x date) in which the measured individual was sampled	eventID	Event

3. Traits

Data for a total number of 180 traits are available in the database, grouped in 10 categories: allocation ratio, architecture, chemistry, dynamics, mechanics, microbial associations, morphology, phenology, physiology and plant size (Figure 3, Table 6). Some of these traits are

commonly used in ecological studies as they are considered key to understand the dynamics of plant populations or the functioning of the ecosystems where they live. Since many of the projects which have led to the collection of trait data collated in FAIRTraits were precisely designed to explore uncharted territories of trait-based ecology, there are also a substantial number of traits which are much less studied (e.g. leaf life span, reproductive phenology, root traits, seed dimensions and shape).

Each trait was modeled as a unique quality x entity combination, which is the formalism followed to describe traits (Mungall et al. 2010, Garnier et al. 2017). For example, root nitrogen content per root dry mass (RNC; a quality) has been measured on three "fine" entities (see below): absorptive roots (abr), transport roots (trr) and the whole root system (wrs). There are thus three quality x entity combinations for this specific quality, i.e. three traits, which are distinguished in the database as RNC_abr, RNC_trr and RNC_wrs, respectively (see Table 6). Overall, qualities (i.e. area, mass, nitrogen content per dry mass) were measured on 17 "fine" entities grouped in 10 "coarse" entities (Table 6). In the example above, fine entities are absorptive roots, transport roots and the whole root system, grouped into the coarse entity "root".

The list of traits grouped by categories, together with the entities on which these were measured is given in Table 6, and a trait map (cf. McCormack et al. 2017) organized by trait categories and coarse entities is shown on Figure 3.

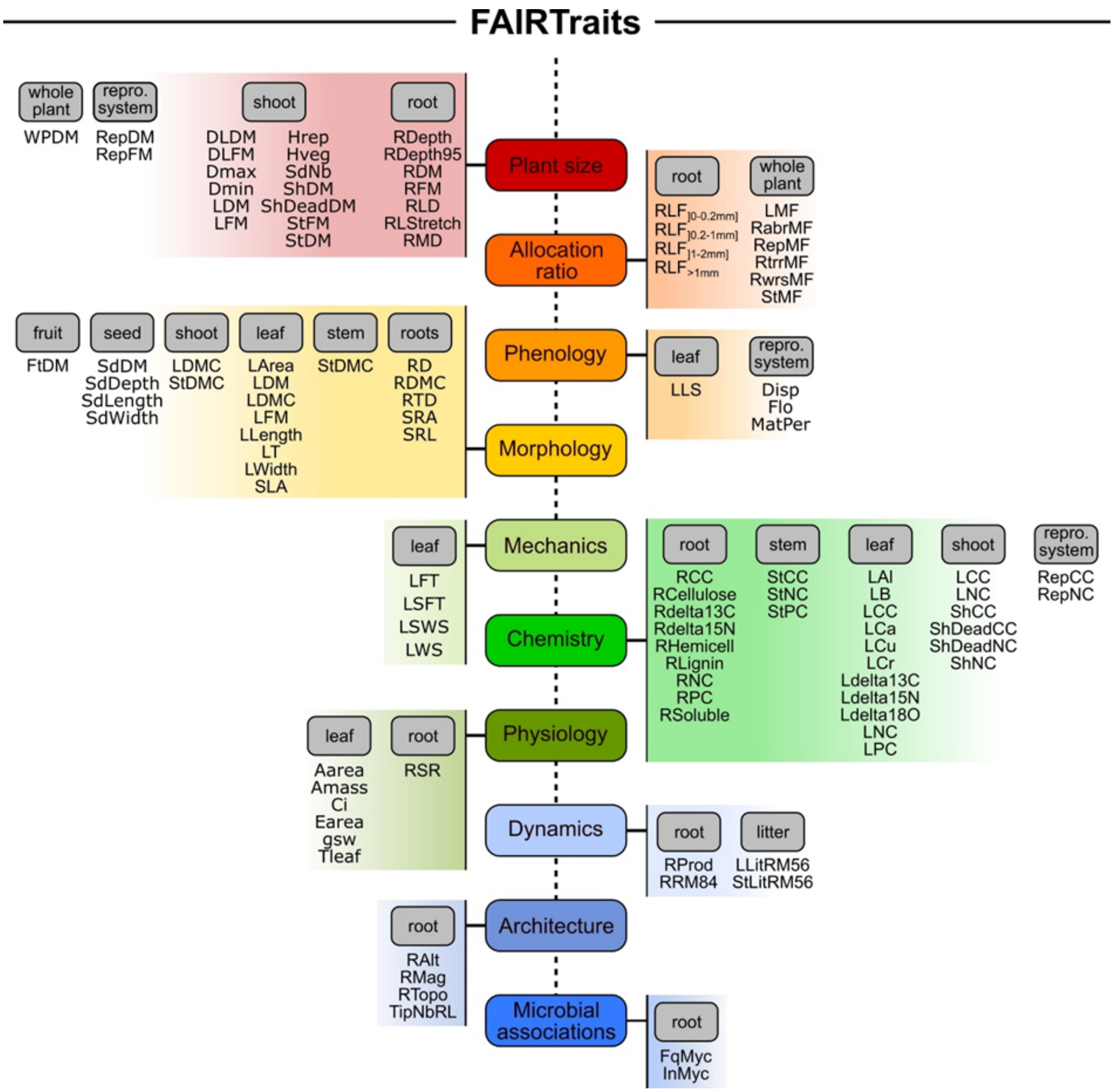

Figure 3. Map of the documented traits in the FAIRTraits database. The traits are grouped into 10 trait categories (plant size, allocation ratio, phenology, morphology, mechanics, chemistry, physiology, dynamics, architecture and microbial associations) and 9 coarse entities (whole plant, reproductive system, fruit, seed, shoot, leaf, stem, litter, root). For the sake of readability, only the most significant traits for each category and entity are shown on the map. See Table 6 for trait abbreviations, details on fine entities, and for the complete list of traits.

Seven variables which are not traits are also present in the database. Five correspond to the environmental conditions in the LI-COR 6400 device chamber during the measurements of gas exchange (“Condition” in Table 6), which are required to interpret properly these data (cf. Ely et al. 2021). The remaining two (“Statistics” in Table 6) are the lower and upper confidence limits of the calculated remaining mass of litter, derived from the fit of an exponential model to the raw data.

Table 6. List of traits with their abbreviations, the coarse and fine entities on which these were measured (entity abbreviation in brackets), and the category to which each trait has been assigned. Traits are sorted first by category, second by coarse entity and third by alphabetic order of trait name. Methods, references and units for measurements are given in the FAIRTraits_TraitValues.txt file. Trait categories build on McCormack et al. (2017).

Trait category	Coarse entity	Fine entity (abbreviation)	Trait name	Trait abbreviation
**Plant size**	whole plant	whole plant (wpl)	Total dry mass of the whole plant	WPDM_wpl
**Plant size**	shoot	mature leaf (mlf), rep. (rsh), veg. (vsh) or whole shoot (wsh)	Total leaf dry mass of the plant	LDM_mlf, LDM_rsh, LDM_vsh, LDM_wsh
**Plant size**	shoot	reproductive shoot (rsh)	Maximum horizontal plant length at reproductive stage	Dmax_rsh
**Plant size**	shoot	reproductive shoot (rsh)	Maximum horizontal plant width at reproductive stage	Dmin_rsh
**Plant size**	shoot	reproductive shoot (rsh)	Reproductive plant height	Hrep_rsh
**Plant size**	shoot	reproductive shoot (rsh)	Total dry mass of reproductive shoots	TshDM_rsh
**Plant size**	shoot	reproductive shoot (rsh)	Total number of seeds produced by the plant	SdNb_rsh
**Plant size**	shoot	vegetative (vsh), reproductive (rsh) or whole shoot (wsh)	Dead shoot dry mass	ShDeadDM_vsh, ShDeadDM_rsh, ShDeadDM_wsh
**Plant size**	shoot	vegetative (vsh), reproductive (rsh) or whole shoot (wsh)	Shoot dry mass	ShDM_vsh, ShDM_rsh, ShDM_wsh
**Plant size**	shoot	vegetative (vsh), reproductive (rsh) or whole shoot (wsh)	Total stem (or sheath) dry mass of the plant	StDM_vsh, StDM_rsh, StDM_wsh
**Plant size**	shoot	vegetative shoot (vsh)	Maximum horizontal plant length at vegetative stage	Dmax_vsh
**Plant size**	shoot	vegetative shoot (vsh)	Maximum horizontal plant width at vegetative stage	Dmin_vsh
**Plant size**	shoot	vegetative shoot (vsh)	Vegetative plant height	Hveg_vsh
**Plant size**	shoot	whole shoot (wsh)	Total dead leaf dry mass of the plant	DLDM_wsh
**Plant size**	shoot	whole shoot (wsh)	Total dead leaf fresh mass of the plant	DLFM_wsh
**Plant size**	shoot	whole shoot (wsh)	Total leaf fresh mass of the plant	LFM_wsh
**Plant size**	shoot	whole shoot (wsh)	Total stem (or sheath) fresh mass of the plant	StFM_wsh
**Plant size**	stem	stem (ste)	Bulb dry mass	BDM_ste
**Plant size**	stem	stem (ste)	Bulb fresh mass	BFM_ste
**Plant size**	root	absorptive (abr) or whole root system (wrs)	Root mass density	RMD_abr, RMD_wrs
**Plant size**	root	absorptive (abr), transport (trr) or whole root system (wrs)	Root length density	RLD_abr, RLD_trr, RLD_wrs
**Plant size**	root	whole root system (wrs)	Rooting depth	RDepth_wrs
**Plant size**	root	whole root system (wrs)	Rooting depth 95%	RDepth95_wrs
**Plant size**	root	whole root system (wrs)	Root dry mass	RDM_wrs
**Plant size**	root	whole root system (wrs)	Root fresh mass	RFM_wrs
**Plant size**	root	whole root system (wrs)	Maximum length of the stretched root system	RLStretch_wrs
**Plant size**	reproductive system	reproductive system (rep)	Total dry mass of reproductive organs	RepDM_rep
**Plant size**	reproductive system	reproductive system (rep)	Total fresh mass of reproductive organs	RepFM_rep
**Allocation ratio**	whole plant	whole plant (wpl)	Leaf mass fraction	LMF_wpl

Trait category	Coarse entity	Fine entity (abbreviation)	Trait name	Trait abbreviation
**Allocation ratio**	whole plant	whole plant (wpl)	Absorptive root mass fraction	RabrMF_wpl
**Allocation ratio**	whole plant	whole plant (wpl)	Reproductive dry mass fraction	RepMF_wpl
**Allocation ratio**	whole plant	whole plant (wpl)	Transport root mass fraction	RtrrMF_wpl
**Allocation ratio**	whole plant	whole plant (wpl)	Whole root system mass fraction	RwrsMF_wpl
**Allocation ratio**	whole plant	whole plant (wpl)	Stem mass fraction	StMF_wpl
**Allocation ratio**	root	whole root system (wrs)	Length fraction of roots with diameter higher than 1 mm	RLF>1mm_wrs
**Allocation ratio**	root	absorptive (abr) or whole root system (wrs)	Length fraction of roots with diameter]0-0.2mm]	RLF]0-0.2mm]_abr, RLF]0-0.2mm]_wrs
**Allocation ratio**	root	absorptive (abr) or whole root system (wrs)	Length fraction of roots with diameter]0.2-1mm]	RLF]0.2-1mm]_abr, RLF]0.2-1mm]_wrs
**Allocation ratio**	root	absorptive roots (abr)	Length fraction of roots with diameter]1-2mm]	RLF]1-2mm]_abr
**Phenology**	leaf cohort	leaf cohort (clf)	Leaf life span	LLS_clf
**Phenology**	reproductive system	reproductive system (rep)	Time of seed dispersal	Disp_rep
**Phenology**	reproductive system	reproductive system (rep)	Onset of flowering	Flo_rep
**Phenology**	reproductive system	reproductive system (rep)	Duration of seed maturation period	MatPer_rep
**Morphology**	shoot	whole shoot (wsh)	Leaf dry matter content	LDMC_wsh
**Morphology**	shoot	whole shoot (wsh)	Stem (or sheath) dry matter content	StDMC_wsh
**Morphology**	leaf	mature leaf (mlf)	Area of a leaf	LArea_mlf
**Morphology**	leaf	mature leaf (mlf)	Dry mass of a leaf	LDM_mlf
**Morphology**	leaf	mature leaf (mlf)	Leaflet width	LfletWidth_mlf
**Morphology**	leaf	mature leaf (mlf)	Fresh mass of a leaf	LFM_mlf
**Morphology**	leaf	mature leaf (mlf)	Leaf length	LLength_mlf
**Morphology**	leaf	mature leaf (mlf)	Leaf lamina thickness excluding midvein	LT_mlf
**Morphology**	leaf	mature leaf (mlf)	Leaf width	LWidth_mlf
**Morphology**	leaf	mature leaf (mlf)	Specific leaf area	SLA_mlf
**Morphology**	leaf	mature leaf (mlf)	Leaf dry matter content	LDMC_mlf
**Morphology**	stem	sheath (she)	Stem dry matter content for graminoid species	StDMC_she
**Morphology**	stem	stem (ste)	Stem dry matter content for non-graminoid species	StDMC_ste
**Morphology**	root	absorptive (abr), transport (trr), deep absorptive roots (dar) or whole root system (wrs)	Root tissue density	RTD_abr, RTD_trr, RTD_dar, RTD_wrs
**Morphology**	root	absorptive (abr), transport (trr) or whole root system (wrs)	Average root diameter	RD_abr, RD_trr, RD_wrs
**Morphology**	root	absorptive (abr), transport (trr) or whole root system (wrs)	Specific root area	SRA_abr, SRA_trr, SRA_wrs
**Morphology**	root	absorptive (abr), transport (trr), deep absorptive roots (dar) or whole root system (wrs)	Specific root length	SRL_abr, SRL_trr, SRL_dar, SRL_wrs
**Morphology**	root	absorptive roots (abr), deep absorptive roots (dar) or whole root system (wrs)	Root dry matter content	RDMC_abr, RDMC_dar, RDMC_wrs
**Morphology**	fruit	fruit (frt)	Fruit dry mass	FtDM_frt

Trait category	Coarse entity	Fine entity (abbreviation)	Trait name	Trait abbreviation
**Morphology**	seed	mature seed (msd)	Seed depth	SdDepth_msd
**Morphology**	seed	mature seed (msd)	Seed dry mass	SdDM_msd
**Morphology**	seed	mature seed (msd)	Seed length	SdLength_msd
**Morphology**	seed	mature seed (msd)	Seed width	SdWidth_msd
**Mechanics**	leaf	mature leaf (mlf)	Leaf tensile strength	LTS_mlf
**Mechanics**	leaf	mature leaf (mlf)	Leaf work to shear	LWS_mlf
**Chemistry**	shoot	rep. (rsh), veg. (vsh) or whole shoot (wsh)	Leaf carbon content per leaf dry mass	LCC_rsh, LCC_vsh, LCC_wsh
**Chemistry**	shoot	rep. (rsh), veg. (vsh) or whole shoot (wsh)	Leaf carbon isotopic fraction	Ldelta13C_rsh, Ldelta13C_vsh, Ldelta13C_wsh
**Chemistry**	shoot	rep. (rsh), veg. (vsh) or whole shoot (wsh)	Leaf nitrogen content per leaf dry mass	LNC_rsh, LNC_vsh, LNC_wsh
**Chemistry**	shoot	rep. (rsh), veg. (vsh) or whole shoot (wsh)	Leaf phosphorus content per leaf dry mass	LPC_rsh, LPC_vsh, LPC_wsh
**Chemistry**	shoot	vegetative (vsh) or reproductive (rsh) shoot	Dead shoot carbon content per dead shoot dry mass	ShDeadCC_vsh, ShDeadCC_rsh
**Chemistry**	shoot	vegetative (vsh) or reproductive (rsh) shoot	Dead shoot nitrogen content per dead shoot dry mass	ShDeadNC_vsh, ShDeadNC_rsh
**Chemistry**	shoot	vegetative (vsh), reproductive (rsh) or whole shoot (wsh)	Stem (or sheath) carbon content per stem (or sheath) dry mass	StCC_vsh, StCC_rsh, StCC_wsh
**Chemistry**	shoot	vegetative (vsh), reproductive (rsh) or whole shoot (wsh)	Stem (or sheath) nitrogen content per stem (or sheath) dry mass	StNC_vsh, StNC_rsh, StNC_wsh
**Chemistry**	shoot	vegetative (vsh), reproductive (rsh) or whole shoot (wsh)	Stem (or sheath) phosphorus content per stem (or sheath) dry mass	StPC_vsh, StPC_rsh, StPC_wsh
**Chemistry**	shoot	vegetative shoot (vsh)	Shoot carbon content per shoot dry mass	ShCC_vsh
**Chemistry**	shoot	vegetative shoot (vsh)	Shoot nitrogen content per shoot dry mass	ShNC_vsh
**Chemistry**	leaf	mature leaf (mlf)	Leaf aluminum content per leaf dry mass	LAl_mlf
**Chemistry**	leaf	mature leaf (mlf)	Leaf boron content per leaf dry mass	LB_mlf
**Chemistry**	leaf	mature leaf (mlf)	Leaf calcium content per leaf dry mass	LCa_mlf
**Chemistry**	leaf	mature leaf (mlf)	Leaf chromium content per leaf dry mass	LCr_mlf
**Chemistry**	leaf	mature leaf (mlf)	Leaf copper content per leaf dry mass	LCu_mlf
**Chemistry**	leaf	mature leaf (mlf)	Leaf nitrogen isotopic fraction	Ldelta15N_mlf
**Chemistry**	leaf	mature leaf (mlf)	Leaf oxygen isotopic fraction	Ldelta18O_mlf
**Chemistry**	leaf	mature leaf (mlf)	Leaf iron content per leaf dry mass	LFe_mlf
**Chemistry**	leaf	mature leaf (mlf)	Leaf potassium content per leaf dry mass	LK_mlf
**Chemistry**	leaf	mature leaf (mlf)	Leaf magnesium content per leaf dry mass	LMg_mlf
**Chemistry**	leaf	mature leaf (mlf)	Leaf manganese content per leaf dry mass	LMn_mlf
**Chemistry**	leaf	mature leaf (mlf)	Leaf sodium content per leaf dry mass	LNa_mlf
**Chemistry**	leaf	mature leaf (mlf)	Leaf nickel content per leaf dry mass	LNi_mlf

Trait category	Coarse entity	Fine entity (abbreviation)	Trait name	Trait abbreviation
**Chemistry**	leaf	mature leaf (mlf)	Leaf lead content per leaf dry mass	LPb_mlf
**Chemistry**	leaf	mature leaf (mlf)	Leaf sulfur content per leaf dry mass	LS_mlf
**Chemistry**	leaf	mature leaf (mlf)	Leaf strontium content per leaf dry mass	LSr_mlf
**Chemistry**	leaf	mature leaf (mlf)	Leaf titanium content per leaf dry mass	LTi_mlf
**Chemistry**	leaf	mature leaf (mlf)	Leaf zinc content per leaf dry mass	LZn_mlf
**Chemistry**	leaf	mature leaf (mlf),	Leaf carbon content per leaf dry mass	LCC_mlf
**Chemistry**	leaf	mature leaf (mlf)	Leaf carbon isotopic fraction	Ldelta13C_mlf
**Chemistry**	leaf	mature leaf (mlf)	Leaf nitrogen content per leaf dry mass	LNC_mlf
**Chemistry**	leaf	mature leaf (mlf)	Leaf phosphorus content per leaf dry mass	LPC_mlf
**Chemistry**	stem	sheath (she)	Stem carbon content per leaf dry mass for graminoid species	StCC_she
**Chemistry**	stem	sheath (she)	Stem nitrogen content per leaf dry mass for graminoid species	StNC_she
**Chemistry**	stem	stem (ste)	Stem carbon content per leaf dry mass for non-graminoid species	StCC_ste
**Chemistry**	stem	stem (ste)	Stem nitrogen content per leaf dry mass for non-graminoid species	StNC_ste
**Chemistry**	root	absorptive (abr), transport (trr) or whole root system (wrs)	Root nitrogen content per root dry mass	RNC_abr, RNC_trr, RNC_wrs
**Chemistry**	root	absorptive (abr), transport (trr) or whole root system (wrs)	Root carbon content per root dry mass	RCC_abr, RCC_trr, RCC_wrs
**Chemistry**	root	absorptive roots (abr)	Root cellulose content per root dry mass	RCellulose_abr
**Chemistry**	root	absorptive roots (abr)	Root carbon isotopic fraction	Rdelta13C_abr
**Chemistry**	root	absorptive roots (abr)	Root nitrogen isotopic fraction	Rdelta15N_abr
**Chemistry**	root	absorptive roots (abr)	Root hemicellulose content per root dry mass	RHemicell_abr
**Chemistry**	root	absorptive roots (abr)	Root lignin content per root dry mass	RLignin_abr
**Chemistry**	root	absorptive roots (abr)	Root water-soluble compounds content per root dry mass	RSoluble_abr
**Chemistry**	root	whole root system (wrs)	Root phosphorus content per root dry mass	RPC_wrs
**Chemistry**	reproductive system	reproductive system (rep)	Reproductive organ carbon content per reproductive organ dry mass	RepCC_rep
**Chemistry**	reproductive system	reproductive system (rep)	Reproductive organ nitrogen content per reproductive organ dry mass	RepNC_rep
**Physiology**	leaf	mature leaf (mlf)	Net CO_2 exchange per leaf area	A_{area}_mlf
**Physiology**	leaf	mature leaf (mlf)	Net CO_2 exchange per leaf dry mass	A_{mass}_mlf

Trait category	Coarse entity	Fine entity (abbreviation)	Trait name	Trait abbreviation
**Physiology**	leaf	mature leaf (mlf)	Intercellular CO_2 concentration in air	Ci_mlf
**Physiology**	leaf	mature leaf (mlf)	Transpiration rate of H_2O per leaf area	E_{area}_mlf
**Physiology**	leaf	mature leaf (mlf)	Stomatal conductance to water vapor per leaf area	g_{sw}_mlf
**Physiology**	leaf	mature leaf (mlf)	Leaf surface temperature	Tleaf_mlf
**Physiology**	root	absorptive roots (abr)	Root specific respiration	RSR_abr
**Dynamics**	root	absorptive roots (abr)	Root productivity	RProd_abr
**Dynamics**	root	absorptive roots (abr)	Root remaining mass	RRM84_abr
**Dynamics**	litter	leaf litter (llt)	Leaf litter remaining mass	LLitRM56_llt
**Dynamics**	litter	leaf litter (llt)	Leaf litter remaining mass calculated	LLitRM56c_llt
**Dynamics**	litter	stem litter (stl)	Stem litter remaining mass	StLitRM56_stl
**Architecture**	root	whole root system (wrs)	Altitude of the whole root system	RAlt_wrs
**Architecture**	root	whole root system (wrs)	Magnitude of the whole root system	RMag_wrs
**Architecture**	root	whole root system (wrs)	Root topological index	RTopo_wrs
**Architecture**	root	whole root system (wrs)	Number of root tips per unit of root length of the whole root system	TipNbRL_wrs
**Microbial associations**	root	absorptive roots (abr)	Root mycorrhizal colonization frequency	FqMyc_abr
**Microbial associations**	root	absorptive roots (abr)	Root mycorrhizal colonization intensity	InMyc_abr
**Condition**	condition	chamber condition (chc)	CO_2 concentration in wet air inside chamber	CO_2s_chc
**Condition**	condition	chamber condition (chc)	Atmospheric pressure	P_{atm}_chc
**Condition**	condition	chamber condition (chc)	In-chamber photosynthetic flux density (PPFD) incident on the leaf, quanta per area	Q_{in}_chc
**Condition**	condition	chamber condition (chc)	Relative humidity of air inside the chamber	RHs_chc
**Condition**	condition	chamber condition (chc)	Time of day for gas exchange measurement	timeOfDay_chc
**Statistics**	litter	leaf litter (llt)	Higher confidence limit of calculated leaf litter remaining mass	HiLLitRM56c_llt
**Statistics**	litter	leaf litter (llt)	Lower confidence limit of calculated leaf litter remaining mass	LoLLitRM56c_llt

Figure 4 shows the number of records in the database organized by life form and taxonomy (panels a-c) and on a trait perspective bases (panels d-f). The majority of records concerns herbaceous species (hemicryptophytes and therophytes), and Poaceace, Fabaceae and Asteraceae were the three families with the largest number of records. As is the case for the number of populations (cf. Figure 2b in section Class II.B.2.a.), the two Poaceae *Bromus erectus* and

Brachypodium phoenicoides are the two species with the largest number of records, followed by the Rosaceae *Potentilla neumanniana*.

Traits pertaining to organ morphology (leaf area, mass, root diameter and specific length, leaf stem and root dry matter content, etc.) are the most represented traits in the database, followed by size-related traits (plant height, horizontal dimensions [Figure 4f], rooting depth, etc.) and chemical composition, including leaf carbon isotopic ratio (Figure 4d,f). We also provide data on several physiological traits: leaf gas exchange (net photosynthesis, transpiration, intrinsic water-use efficiency) for 60 species (70 populations), root respiration for 16 species, leaf litter and root decomposition for 42 species and 33 species, respectively. Flowering onset and time of seed dispersal (reproductive phenology) are available for 160 and 164 species, respectively. While leaf and shoot traits represent the majority of the records, root and seed traits also substantially contribute to the database (Figure 4e).

The number of records, minimum, maximum and distribution of trait values together with their unit are given in Figure 5.

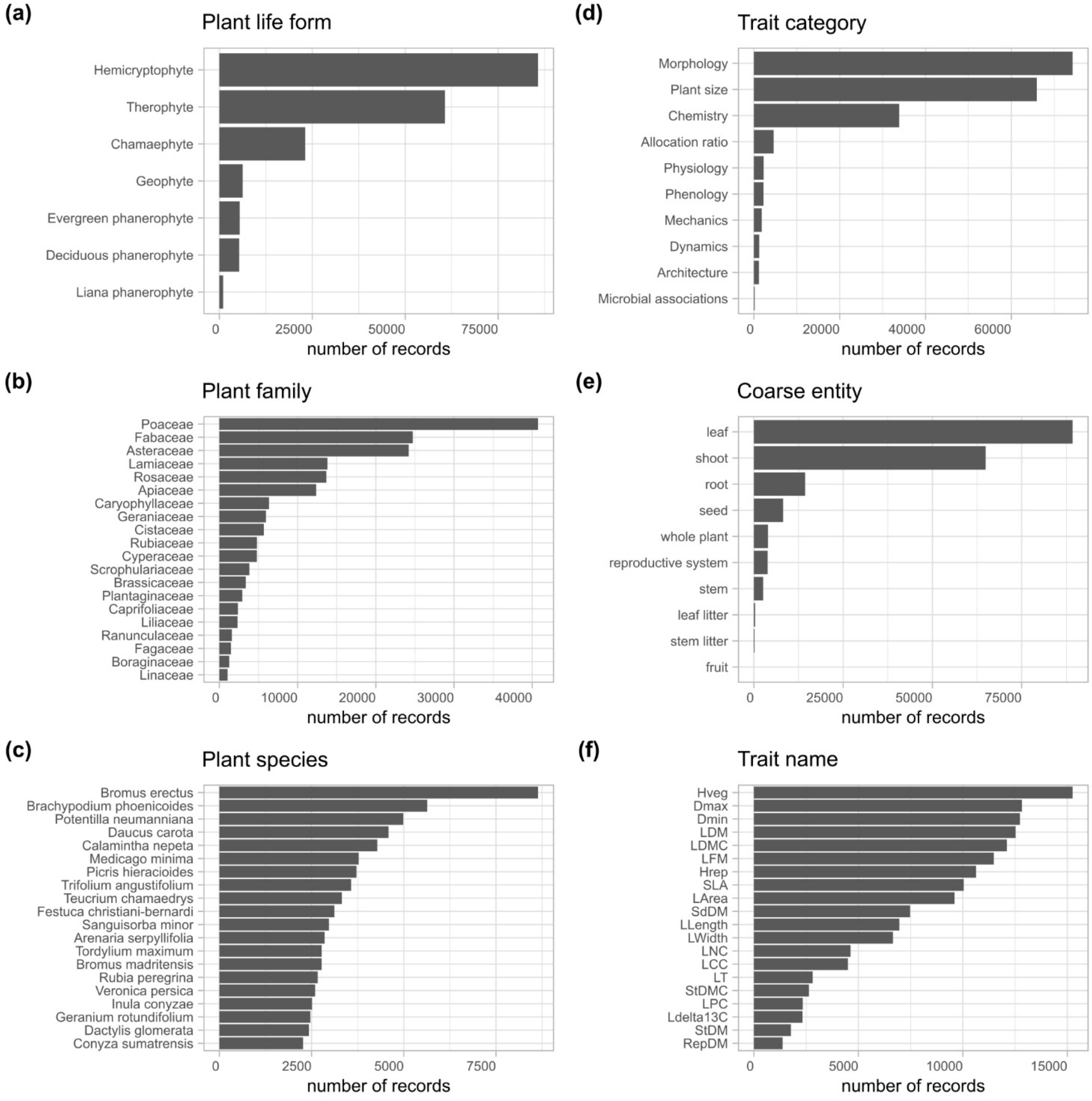

Figure 4. Number of records in the FAIRTraits database organized by (a) plant life form *sensu* Raunkiær (1934), (b) botanical family for the 20 most represented families, (c) species for the 20 most represented species, (d) trait category, (e) coarse entity and (f) trait name for the 20 most documented traits (*Hveg* : vegetative plant height ; *Dmax* : maximum horizontal plant length when plant in vegetative stage ; *Dmin* : maximum horizontal plant width when plant in vegetative stage; *LDM* : dry mass of a leaf ; *LDMC* : leaf dry matter content ; *LFM* : fresh mass of a leaf; *Hrep* : reproductive plant height ; *SLA* : specific leaf area ; *LArea* : area of a leaf ; *SdDM* : seed dry mass ; *LLength* : leaf length ; *LWidth* : leaf width ; *LNC* : leaf nitrogen content per leaf dry mass ; *LCC* : leaf carbon content per leaf dry mass ; *LT* : leaf lamina thickness excluding midvein ; *StDMC* : stem or sheath dry matter content ; *LPC* : leaf phosphorus content per leaf dry mass ; *Ldelta13C* : leaf carbon isotopic fraction ; *StDM* : total stem or sheath dry mass of the plant; *RepDM* : total dry mass of reproductive organs). See Table 6 for trait abbreviations, details on fine entities, and the complete list of traits.

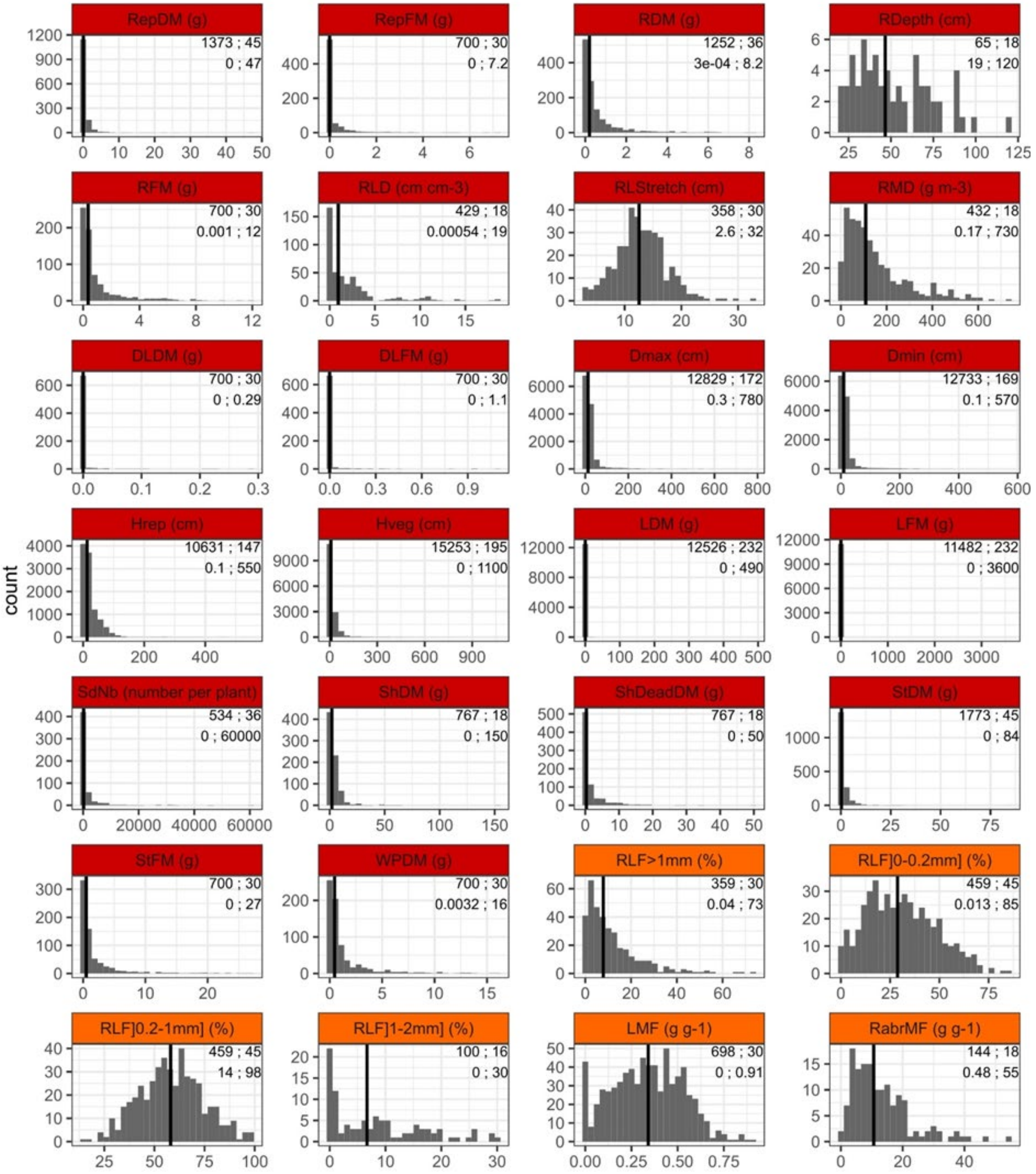

Figure 5. Frequency distributions of trait values for the 100 most documented traits in the FAIRTraits database. Units are shown next to the trait abbreviations presented in Table 6. Colors indicate the trait category as presented in Figure 3 (plant size, allocation ratio, phenology, morphology, mechanics, chemistry, physiology, dynamics, architecture and microbial associations). The number of records and number of species (upper line), and minimum and maximum values (lower line) are indicated for each trait in each panel. Vertical lines indicate the median values of the traits.

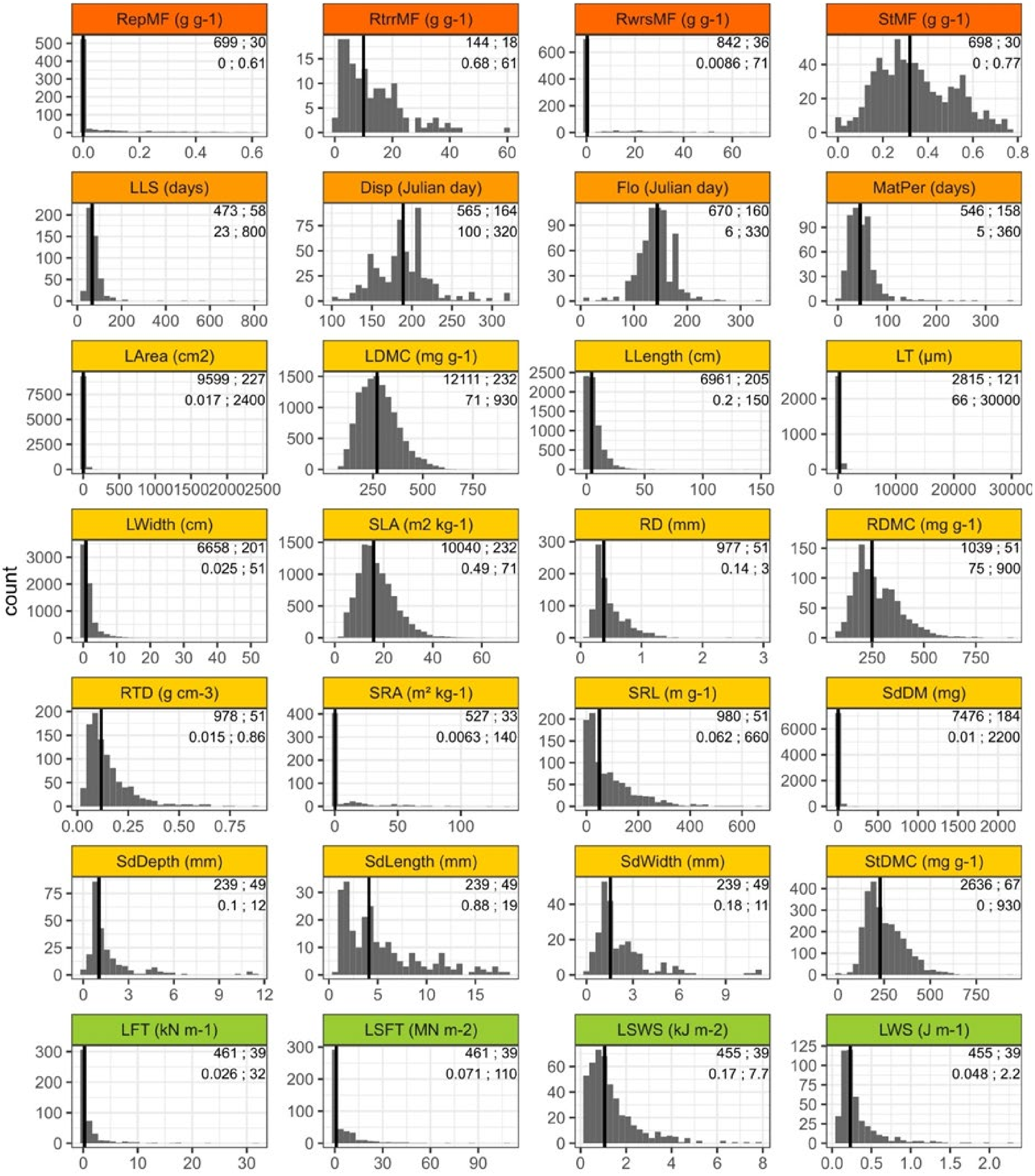

Figure 5. Continued.

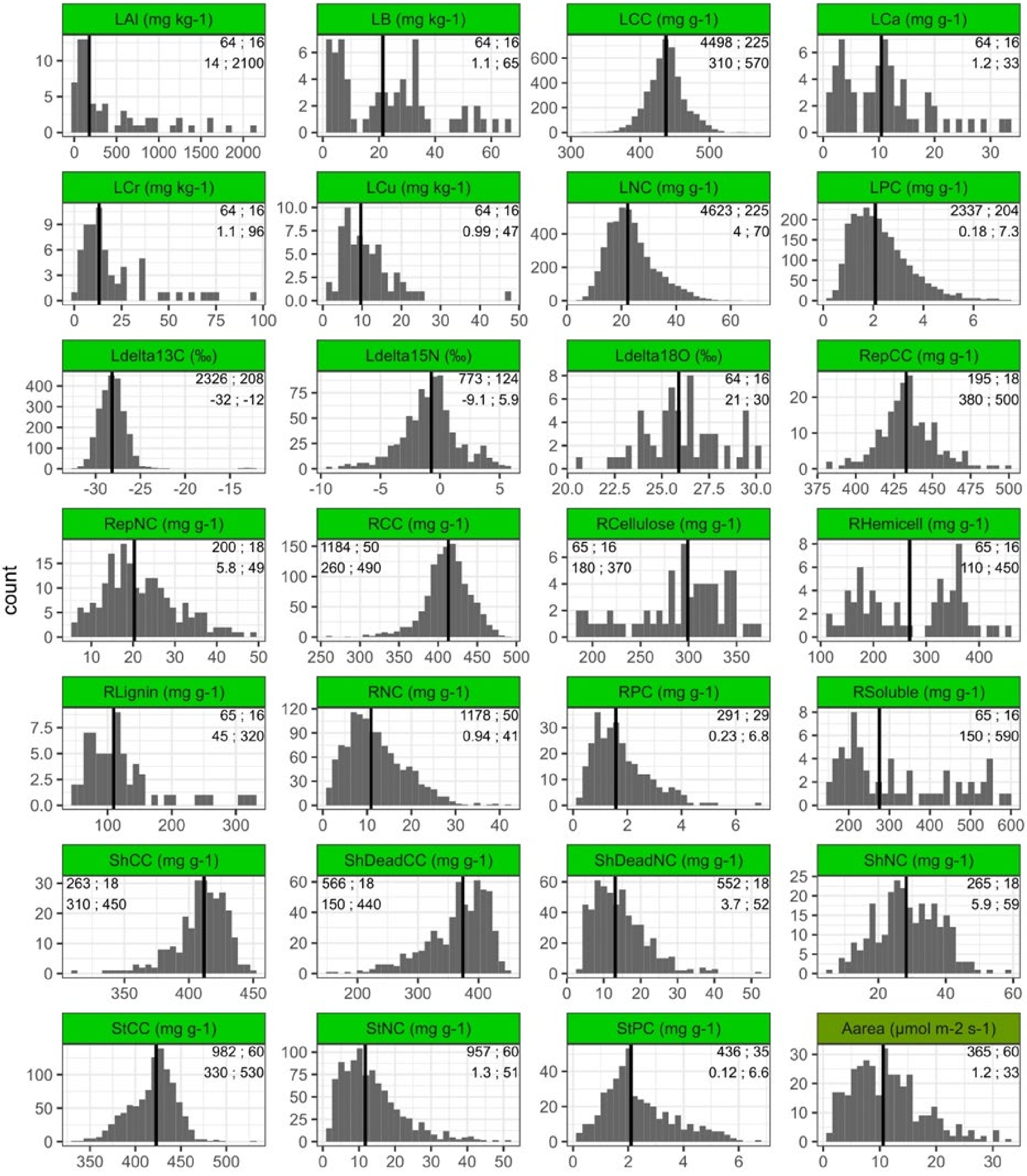

Figure 5. Continued.

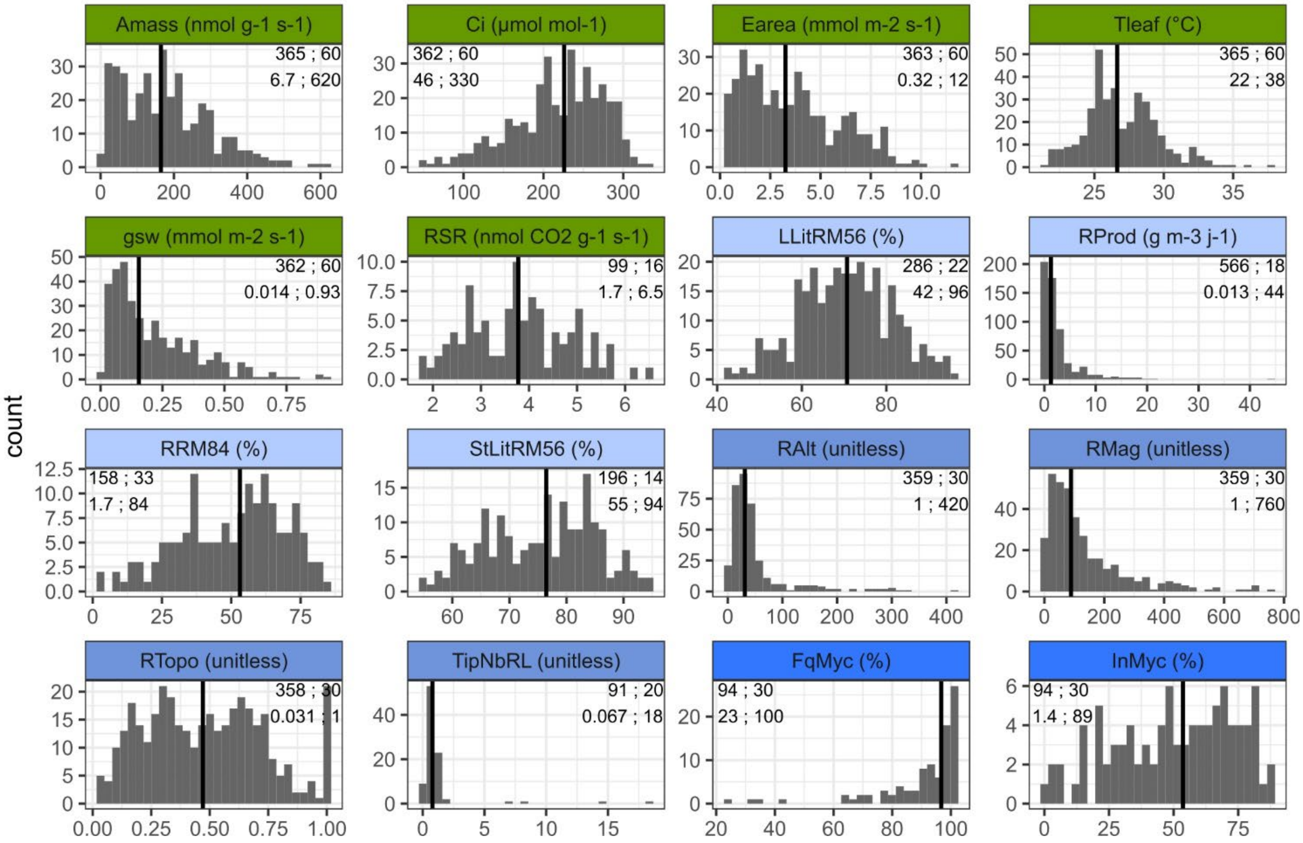

Figure 5. Continued.

4. Environmental data

Trait values not only depend on the taxa on which they are determined, but also on the environmental conditions in which the individuals representing these taxa are sampled (Violle et al. 2007, Garnier et al. 2016, Shipley et al. 2016, Anderegg 2023 for references and discussions). To describe the environmental context in which the FAIRTraits data were collected, we provide information on climatic variables at the site level taken from the meteorological station closest to each site (cf. Table 3 and Figure 1 for climatic diagrams, in section Class II.B.1.), and on treatments, disturbance (type, intensity and return interval) and soil characteristics at the plot level (see section Class III.B.2.a. above for details on the design). The list of environmental variables available in the database is given in Table 7. Since most of these environmental variables are not defined in the Darwin Core standard, these data are not available as a Darwin Core archive. They are archived as txt files in the InDoRES repository.

Table 7. List of variables in the three files describing environmental data: FAIRTraits_MeteoSitesYear.txt (annMet) and FAIRTraits_MeteoSitesMonth.txt files (monMet) and FAIRTraits_LocalEnvironment.txt (localEnv) files. In this latter file, soil type, treatments and a qualitative description of resource level (nutrient or water) are given at the "traitPlot" level, while soil properties were measured on a subset of "soilPlots" (section Class B.2.a. for details). Methods, references and units are given in the FAIRTraits_DescripEnvVariables.txt file.

Name of variable	File	Name of variable
**siteName**	monMet, annMet & localEnv	Name of site
**siteLatitude**	monMet & annMet	Latitude of site
**siteLongitude**	monMet & annMet	Longitude of site
**siteMinAltitude**	monMet & annMet	Minimum altitude of site
**siteMaxAltitude**	monMet & annMet	Maximum altitude of site
**siteMeteoStationName**	monMet & annMet	Name of meteorological station for site
**siteMeteoStationID**	monMet & annMet	Identifier of meteorological station for site
**siteDistMeteoStation**	monMet & annMet	Distance from site to meteorological station
**siteMeteoStationLatitude**	monMet & annMet	Latitude of meteorological station for site
**siteMeteoStationLongitude**	monMet & annMet	Longitude of meteorological station for site
**siteMeteoStationAltitude**	monMet & annMet	Altitude of meteorological station for site
**siteMeteoStationCalculationPeriod**	monMet & annMet	Calculation period for meteorological data
**siteMeteoStationMAT**	annMet	Mean annual temperature from meteorological station
**siteMeteoStationMColdM**	annMet	Mean temperature of the coldest month from meteorological station
**siteMeteoStationMWarM**	annMet	Mean temperature of the warmest month from meteorological station
**siteMeteoStationMAP**	annMet	Mean annual precipitation from meteorological station
**siteMeteoStationETP**	annMet	Mean annual potential evaporation from meteorological station
**siteMeteoStationMonth**	monMet	Month of data record from the meteorological station
**siteMeteoStationMMT**	monMet	Mean monthly temperature from meteorological station
**siteMeteoStationMMMaxT**	monMet	Mean monthly maximal temperature from meteorological station
**siteMeteoStationMMMinT**	monMet	Mean monthly minimal temperature from meteorological station
**siteMeteoStationMMP**	monMet	Mean monthly precipitation from meteorological station
**siteMeteoStationMMETP**	monMet	Mean monthly potential evaporation from meteorological station
**traitPlotTreatment**	localEnv	Identification of treatment
**traitPlotTreatmentDescription**	localEnv	Short description of treatment
**traitPlotRefTreatmentDescription**	localEnv	Literature reference for detailed description of treatment
**traitPlotDisturbanceType**	localEnv	Type of disturbance in plot (grazing, cultivation, etc.)
**traitPlotDisturbanceIntensity**	localEnv	Disturbance intensity in plot
**traitPlotDisturbanceReturnInterval**	localEnv	Return time interval of disturbance in plot
**traitPlotFertilization**	localEnv	Level of fertilization in plot
**traitPlotSoilType**	localEnv	Type of soil in plot
**soilPlotID**	localEnv	Identification code of the plot sampled for soil measurements
**soilPlotLatitude**	localEnv	Latitude of soil plot
**soilPlotLongitude**	localEnv	Longitude of soil plot
**soilPlotAltitude**	localEnv	Altitude of soil plot
**soilPlotPeriodSoilAnalyses**	localEnv	Time period when soil samples were collected for analyses

Name of variable	File	Name of variable
**soilPlotSoilLayerAnalyzed**	localEnv	Minimum and maximum depths of soil sampled for analyses
**soilPlotHumidity**	localEnv	Humidity of fresh soil
**soilPlotClay**	localEnv	Clay fraction of soil (particle size < 2 µm)
**soilPlotSilt**	localEnv	Silt fraction of soil (particle size between 2 and 50 µm)
**soilPlotSand**	localEnv	Sand fraction of soil (particle size > 50 µm)
**soilPlotCaCO3**	localEnv	Total lime content of soil per mass
**soilPlotCorg**	localEnv	Organic carbon content of soil per mass
**soilPlotNTotal**	localEnv	Total (organic + mineral) nitrogen content of soil per mass
**soilPlotCNRatio**	localEnv	Ratio of organic content to total nitrogen content of soil
**soilPlotOrgMat**	localEnv	Organic matter content of soil per unit mass
**soilPlotpH**	localEnv	Soil pH in water
**soilPlotPOlsen**	localEnv	Extractable phosphorus content of soil per mass
**soilPlotCEC**	localEnv	Cation-exchange capacity of soil per mass
**soilPlotAggregationMethod**	localEnv	Method used to infer soil values for plots with unavailable data

Class V. Supplemental descriptors

A. Data acquisition

1. Location of completed data forms

Paper data sheets still available from the original field data collections are stored by the ECOPAR research group in the premises of the Centre d'Écologie Fonctionnelle et Évolutive (CNRS Campus: 1919 route de Mende, 34293 Montpellier Cedex, France).

2. Data entry verification procedures

Field and laboratory data were first digitized by the person(s) in charge of the observations and measurements in spreadsheet files of different formats. Subsets of data were checked and cleaned prior to analyses conducted in the context of the different research projects. Data on reproductive phenology was first consolidated across the whole database by Jules Segrestin (see Segrestin et al. 2018). The current version of FAIRTraits was compiled and reviewed by Eric Garnier, Léo Delalandre, Karim Barkaoui and Jules Segrestin.

B. Quality assurance/quality control procedures

The database underwent extensive quality assurance and control processes before publication. Trait names, taxon names, plot inventories, unexpected missing data, and inconsistencies in methods were corrected during the structuring process, which converted raw data files into the

basic elements described in Table 1 (section Class I.B.). These steps are described in detail in Table 8 (Section Class V.C.).

The final version of FAIRTraits includes values for 180 traits and seven additional variables (cf. section Class IV.B.3.) measured across 1 to 240 species. Error detection for each trait and species followed a two-step approach:

First for species with sufficient replicates ($n > 4$), we tested the consistency of units by assessing whether the distribution of trait values across all replicates followed a unimodal pattern. This was done using the *dip.test* function from the *diptest* R package (Maechler, 2024). For distributions with significant results ($P < 0.001$), we manually inspected the data and corrected any discrepancies in unit expression. This process identified cases where trait values collected during a given field campaign (e.g., a sampling season at one site) were systematically recorded using a unit different from that used during other campaign(s) (e.g., meters instead of centimeters for plant height). In such cases, all replicate trait values for all species recorded during the campaign were corrected accordingly.

Second, errors in individual observations were identified using the Grubbs' test for outlier detection (*grubbs.test* function in the *outliers* R package; Komsta, 2022). The test was applied separately to each trait and species with at least five replicates. For significant results ($P < 0.05$), we manually inspected the data. Obvious errors were corrected, while values determined to be true outliers were conservatively removed from the database following recommendations from Keller et al. (2023). Size related traits (total, or above-ground, plant biomass, etc.) and allocation ratios constitute special cases however. These traits can actually vary greatly in the field (e.g., between young and old individuals), reflecting relevant biological variation rather than measurement errors. In this case, the corresponding trait values were retained in the database.

C. Computer programs and data-processing algorithms

The re-structuring of data from native files to the database presented here was performed using the software R (R Core Team 2025). All the scripts (Delalandre 2025) used for processing the data are available on Zenodo (https://doi.org/10.5281/zenodo.17073323). They are described in Table 8.

Table 8 Description of the scripts used to process the data from the raw data files to the FAIRTraits_TraitValues.txt file.

Name of script	Actions performed
**core_1_import_data.R**	This script imports raw trait data from multiple excel files and writes a single txt file with one row corresponding to one record (i.e., one trait measured on one individual at one time)
**core_2_generate_core.R**	This script imports the previously generated txt file (containing one record per row), updates the values taken in some columns (Plot, Treatment), and corrects typos.
**core_3_add_MoFTraits_to_core.R**	This script updates trait names and adds information on trait measurement and sampling method to the core of the database, using an input file constructed manually. This file is called MoFTraits, MoF being the abbreviation of Measurement or Fact, which is defined in the Ecological Trait-data Standard (Schneider et al. 2019).
**core_4_add_plot_info_to_core.R**	This script adds information on altitude, longitude, and latitude, to the core of the database. It uses as additional inputs manually-constructed files containing environmental information, and mapping information from native names of plots, treatments, and sites, to the names used in the present database.
**core_5_add_occurrence_to_core.R**	This script generates occurrenceIDs, defined at three levels: the level of data record (verbatimOccurrenceID); the level of the replicate on which a specific data record was taken (verbatimOccurrenceId_sample); the level of the population (species x site x plot x treatment x date) in which the measured individual was sampled.
**core_6_DarwinCore_mapping.R**	This script changes column names of the database to Darwin Core names, as described in Table 4, section IV.B.1.
**quality_check_non_numeric_fields.R**	Runs all the previously described scripts.
**taxon.R**	Quality control of non-numeric fields. Quality control for numeric fields (i.e., trait data) is presented in section V.B. This script verifies the class of each column, identifies empty fields, checks for duplicated ID names, and checks the correspondence between multiple fields, within and across data tables.

D. Archiving

1. Archival procedures

Data and metadata on taxa and traits values are available on the GBIF infrastructure (https://www.gbif.org/fr/) as "Checklist" and "Occurrence" Darwin Core archives respectively (Table 9). The Checklist archive is composed of two files corresponding to the Taxon Core and the Plinian Core extension, while the Occurrence archive is composed of three files corresponding to the Occurrence Core and to the Trait measurement score and Trait descriptor extensions. These cores and extensions were required to map the FAIRTraits data to the Darwin Core standard. In GBIF, metadata are available as rtf, xml and eml files. Since environmental data could not be mapped easily onto the Darwin Core standard, these data are made available in the InDoRES repository (https://www.indores.fr/) as txt files (Table 9). For these data, metadata are available as a README file on InDoRES, and under several formats in the Cat.InDoRES metadata catalog (https://cat.indores.fr/geonetwork/srv/eng/catalog.search#/home).

The associated R code (Delalandre 2025) used to process the files (Table 8 in section Class V.C.) is available in Zenodo at https://doi.org/10.5281/zenodo.17073323.

Table 9 Names and doi of the FAIRTraits files in the GBIF and InDoRES archives. Note that in the GBIF archives, the FAIRTraits_Taxon.txt and FAIRTraits_TraitValues.txt elements are respectively split into two and three files, corresponding to the Darwin Core extensions used to map the fields of the database. In the InDoRES archive, names and number of files are unchanged.

Basic element	Type	Repository	Name of the archive	doi of the archive	Component(s) of the archive
**FAIRTraits_Authors.txt**	Metadata	GBIF	dwca-fairtraits-v1.0	10.15468/r5kj3q	rtf-faitraits-v1.0.rtf and eml and xml files
		InDoRES	dataverse_files	https://data.indores.fr/dataset.xhtml?persistentId=doi:10.48579/PRO/0IYLBV	README.md
**FAIRTraits_Projects.txt**	Metadata	GBIF	dwca-fairtraits-v1.0	10.15468/r5kj3q	rtf-faitraits-v1.0.rtf and eml and xml files
		InDoRES	dataverse_files	https://data.indores.fr/dataset.xhtml?persistentId=doi:10.48579/PRO/0IYLBV	FAIRTraits_Projects.txt
**FAIRTraits_Taxon.txt**	Data	GBIF	dwca-fairtraits_taxa-v1.0	10.15468/sycrup	taxon.txt
			dwca-fairtraits_taxa-v1.0	https://ipt-uat.gbif.fr/resource?r=fairtraits_taxa	pliniancore.txt
**FAIRTraits_TraitValues.txt**	Data	GBIF	dwca-fairtraits-v1.0	10.15468/r5kj3q	measurementscore.txt
			dwca-fairtraits-v1.0	10.15468/r5kj3q	measurementtrait.txt
			dwca-fairtraits-v1.0	10.15468/r5kj3q	occurrence.txt
**FAIRTraits_MeteoSitesMonth.txt**	Data	InDoRES	dataverse_files	https://data.indores.fr/dataset.xhtml?persistentId=doi:10.48579/PRO/0IYLBV	FAIRTraits_MeteoSitesMonth.txt
**FAIRTraits_MeteoSitesYear.txt**	Data	InDoRES	dataverse_files	https://data.indores.fr/dataset.xhtml?persistentId=doi:10.48579/PRO/0IYLBV	FAIRTraits_MeteoSitesYear.txt
**FAIRTraits_LocalEnvironment.txt**	Data	InDoRES	dataverse_files	https://data.indores.fr/dataset.xhtml?persistentId=doi:10.48579/PRO/0IYLBV	FAIRTraits_LocalEnvironment.txt
**FAIRTraits_PlotCorrespondence.txt**	Data	InDoRES	dataverse_files	https://data.indores.fr/dataset.xhtml?persistentId=doi:10.48579/PRO/0IYLBV	FAIRTraits_PlotCorrespondence.txt
**FAIRTraits_DescripEnvVariable.txt**	Metadata	InDoRES	dataverse_files	https://data.indores.fr/dataset.xhtml?persistentId=doi:10.48579/PRO/0IYLBV	FAIRTraits_DescripEnvVariables.txt
**FAIRTraits_PublishedPapers.txt**	Metadata	InDoRES	dataverse_files	https://data.indores.fr/dataset.xhtml?persistentId=doi:10.48579/PRO/0IYLBV	FAIRTraits_PublishedPapers.txt

2. Redundant archival sites

Cross references among files deposited in the GBIF and InDoRES repositories are insured *via* proper mention of the respective identifiers (doi) attributed to the different archives.

E. Publications and results

Subsets of the database have been used in studies leading to the following publications. Associated research projects are given in brackets (see section Class II.A.6.). This information is available in the FAIRTraits_PublishedPapers.txt file.

An "" in front of the name of the first author indicates that the subset has been combined with data from other origins in broad compilations and/or analyses.*

Barkaoui, K., Bernard-Verdier, M., Navas, M.-L. (2013). Questioning the reliability of the point intercept method for assessing community functional structure in low-productive and highly diverse Mediterranean grasslands. Folia Geobotanica, 48 (3): 393-414. 10.1007/s12224-013-9172-2 (DivHerbe).

Barkaoui, K., Roumet, C., & Volaire, F. (2016). Mean root trait more than root trait diversity determines drought resilience in native and cultivated Mediterranean grass mixtures. Agriculture, Ecosystems & Environment, 231: 122–132. 10.1016/j.agee.2016.06.035 (O2LA).

Bernard-Verdier, M., Flores, O., Navas, M.-L., Garnier E. (2013). Partitioning phylogenetic and functional diversity into alpha and beta components along an environmental gradient in a Mediterranean rangeland. Journal of Vegetation Science, 24: 877–889. 10.1111/jvs.12048 (DivHerbe).

Bernard-Verdier, M., Navas, M.-L., Vellend, M., Violle, C., Fayolle, A. & Garnier, E. (2012). Community assembly along a soil depth gradient: contrasting patterns of plant trait convergence and divergence in a Mediterranean rangeland. Journal of Ecology, 100: 1422-1433. 10.1111/1365-2745.12003 (DivHerbe).

Birouste M., Kazakou E. Blanchard A., Roumet C. 2012. Plants traits and decomposition: are the relationships for roots comparable to those for leaves? Annals of Botany, 109: 463-472. 10.1093/aob/mcr297 (RESPIRS).

Bumb, I., Garnier, E., Bastianelli, D., Richarte, J., Bonnal, L., Kazakou, E. (2016). Influence of management regime and harvest date on the forage quality of rangelands plants: the importance of dry matter content. AoB Plants, 8: pwl045. 10.1093/aobpla/plw045 (CASCADE).

Bumb, I., Garnier, E., Coq, S., Nahmani, J., Del Rey Granado, M., Gimenez, O., Kazakou E (2018). Traits determining the digestibility– decomposability relationships in species from Mediterranean rangelands. Annals of Botany, 121(3): 459-469. 10.1093/aob/mcx175 (CASCADE).

Chollet, S., Rambal, S., Fayolle, A., Hubert, D., Foulquié, D., & Garnier, E. (2014). Combined effects of climate, resource availability and plant traits on biomass produced in a Mediterranean rangeland. Ecology, 95: 737-748. 10.1890/13-0751.1 (DivHerbe).

*Cornwell, W.K., Cornelissen, J.H.C., Amatangelo, K., Dorrepaal, E., Eviner, V.T., Godoy, O., Hobbie, S.E. et al. (2008). Plant traits are the dominant control on litter decomposition rates within biomes worldwide. Ecology Letters, 11: 1065-1071. 10.1111/j.1461-0248.2008.01219.x (VISTA).

Cortez, J., Garnier, E., Pérez-Harguindeguy, N., Debussche, M. & Gillon, D. (2007). Plant traits, litter quality and decomposition in a Mediterranean old-field succession. Plant and Soil, 296: 19-34. 10.1007/s11104-007-9285-6 (VISTA).

Delalandre, L., Violle, C., Coq, S., Garnier, E. (2023). Trait-environment relationships depend on species life history. Journal of Vegetation Science, 34(6): e13211. 10.1111/jvs.13211 (FageAnnuals).

*Díaz, S., Kattge, J., Cornelissen, J.H.C., Wright, I.J., Lavorel, S., Dray, S., Reu, B., Kleyer, M., Wirth, C., Prentice, I.C., Garnier, E., et al. (2016). The global spectrum of plant form and function. Nature, 529: 167–171. 10.1038/nature16489 (VISTA).

*Díaz, S., Kattge, J., Cornelissen, J.H.C., Wright, I.J., Lavorel, S., Dray, S., Reu, B., Kleyer, M., Wirth, C., Prentice, I.C., Garnier, E., et al. (2022). The global spectrum of plant form and function: enhanced species-level trait dataset. Scientific Data, 9: 755. 10.1038/s41597-022-01774-9 (VISTA).

Dungan, R. J., Navas, M.-L., Duncan, R. P., et al. (2008). Effects of leaf emergence on leaf lifespan are independent of life form and successional status. Austral Ecology, 33: 932-939. 10.1111/j.1442-9993.2008.01875.x. (MELODY).

Fayolle, A., Violle, C., & Navas, M.-L. (2009). Differential impacts of plant interactions on herbaceous species recruitment: Disentangling factors controlling emergence, survival and growth of seedlings. Oecologia, 159: 817–825. https://doi.org/10.1007/s00442-008-1254-0 (GEOTRAITS)

*Flores O, Garnier E, Wright IJ, Reich PB, Pierce S, Díaz S, Pakeman RJ, Rusch GM, Bernard-Verdier M, Testi B, et al. 2014. An evolutionary perspective on leaf economics: phylogenetics of leaf mass per area in vascular plants. Ecology and Evolution 4: 2799–2811. 10.1002/ece3.1087 (MELODY).

Fort F., Volaire F., Guilioni L., Barkaoui K., Navas M.-L., Roumet C. (2017). Root traits are related to plant water-use among rangeland Mediterranean species. Functional Ecology 31: 1700-1709. 10.1111/1365-2435.12888 (O2LA).

*Fortunel, C., Garnier, E., Joffre, R., Kazakou, E., Quested, H., Grigulis, K., Lavorel, S. et al. (2009). Leaf traits capture the effects of land use changes and climate on litter decomposability of grasslands across Europe. Ecology, 90: 598-611. 10.1890/08-0418.1 (VISTA).

Fortunel, C., Violle, C., Roumet, C., Buatois, B., Navas, M.-L., Garnier, E. (2009). Allocation strategies and seed traits are hardly affected by nitrogen supply in 18 species differing in successional status. Perspectives in Plant Ecology, Evolution and Systematics, 11: 267–283. 10.1016/j.ppees.2009.04.003 (GEOTRAITS).

*Gardarin, A., Garnier, E., Carrère, P., et al. (2014). Plant trait–digestibility relationships across management and climate gradients in permanent grasslands. Journal of Applied Ecology, 51: 1207-1217. 10.1111/1365-2664.12293 (DivHerbe).

Garnier, E., Cortez, J., Billès, G., Navas, M.-L., Roumet, C., Debussche, M., Laurent, G., Blanchard, A., Aubry, D., Bellmann, A., Neill, C. & Toussaint, J.-P. (2004). Plant functional markers capture ecosystem properties during secondary succession. Ecology, 85: 2630-2637. 10.1890/03-0799 (INDIGO).

Garnier, E., Fayolle, A., Navas, M.-L., Damgaard, C., Cruz, P., Hubert, D., Richarte, J., Autran, P., Leurent, C., Violle, C. (2018). Plant demographic and functional responses to management intensification: A long-term study in a Mediterranean rangeland. Journal of Ecology, 106: 1363–1376. 10.1111/1365-2745.12996 (DivHerbe).

Garnier, E., Laurent, G., Bellmann, A., Debain, S., Berthelier, P., Ducout, B., Roumet, C. & Navas, M.-L. (2001). Consistency of species ranking based on functional leaf traits. New Phytologist, 152: 69-83. 10.1046/j.0028-646x.2001.00239.x (MELODY).

*Garnier, E., Lavorel, S., Ansquer, P., Castro, H., Cruz, P., Dolezal, J., Eriksson, O. et al. (2007). Assessing the effects of land use change on plant traits, communities and ecosystem functioning in grasslands: a standardized methodology and lessons from an application to 11 European sites. Annals of Botany, 99: 967-985. 10.1093/aob/mcl215 (VISTA).

Garnier, E., Vile, D., Debain, S., Bottin, L., Laurent, G. & Roumet, C. (2025). Photosynthesis, water-use and nitrogen relate to both plant height and leaf structure in 60 species from the Mediterranean. Functional Ecology 39: 567-582. 10.1111/1365-2435.14737 (MELODY, DynEcoMed)

Garnier, E., Vile, D., Roumet, C., Lavorel, S., Grigulis, K., Navas, M.-L., Lloret, F. (2019). Inter- and intra-specific trait shifts among sites differing in drought conditions at the north western edge of the Mediterranean Region. Flora, 254: 147–160. 10.1016/j.flora.2018.07.009 (DynEcoMed).

Hummel I., Vile D., Violle C., Devaux J., Ricci B., Blanchard A., Garnier E., Roumet C. (2007). Relating root structure and anatomy to whole plant functioning in 14 herbaceous Mediterranean species. New Phytologist 173: 313-321. 10.1111/j.1469-8137.2006.01912.x (GEOTRAITS).

*Kattge, J., Bönisch, G., Díaz, S., Lavorel, S., Prentice, I.C., Leadley, P., Tautenhahn, S., Werner, G.D.A., Aakala, T., Abedi, M., et al. (2020). TRY plant trait database – enhanced coverage and open access. Global Change Biology, 26: 119–188. 10.1111/gcb.14904 (VISTA).

*Kattge, J., Díaz, S., Lavorel, S., Prentice, I.C., Leadley, P., Bönisch, G., Garnier, E. et al. (2011). TRY – a global database of plant traits. Global Change Biology, 17: 2905-2935. 10.1111/j.1365-2486.2011.02451.x (VISTA).

Kazakou, E, Vile, D., Shipley, B., Gallet, C. & Garnier, E. (2006). Co-variations in litter decomposition, leaf traits and plant growth in species from a Mediterranean old-field succession. Functional Ecology, 20: 21-30. 10.1111/j.1365-2435.2006.01080.x (VISTA).

Kazakou, E., Bumb, I., Garnier, E. (2022). Species dominance rather than complementarity drives community digestibility and litter decomposition in species-rich Mediterranean rangelands. Applied Vegetation Science, 25(4): e12685. 10.1111/avsc.12685 (CASCADE).

Kazakou, E., Garnier, E., Navas, M.-L., Roumet, C., Collin, C., Laurent, G. (2007). Components of nutrient residence time and the leaf economics spectrum in species from Mediterranean old-fields differing in successional status. Functional Ecology, 21: 235-245. 10.1111/j.1365-2435.2006.01242.x (VISTA).

Kazakou, E., Gimenez, O., Garnier, E. (2007). Assessing the relative contribution of leaf lifespan and nutrient resorption to mean residence time: an elasticity analysis. Ecology, 88: 1857-1863. 10.1890/06-1352.1 (VISTA).

Kazakou, E., Violle, C., Roumet, C., Navas, M.-L., Vile, D., Kattge, J., Garnier, E. (2014). Are trait-based species rankings consistent across data sets and spatial scales? Journal of Vegetation Science, 25: 235–247. 10.1111/jvs.12066 (VISTA, GEOTRAITS).

Kazakou, E., Violle, C., Roumet, C., Pintor, C., Gimenez, O., Garnier, E. (2009). Litter quality and decomposability of species from a Mediterranean succession depend on leaf traits but not on nitrogen supply. Annals of Botany, 104: 1151–116. 10.1093/aob/mcp202 (VISTA).

*Lavorel, S., de Bello, F., Grigulis, K., Lepš, J., Garnier, E., Castro, H., Dolezal, J., Godolets, C., Quétier, F. & Thébault, A. (2011). Response of herbaceous vegetation functional diversity to land use change across five sites in Europe and Israel. Israel Journal of Ecology and Evolution, 57: 53-72. 10.1560/IJEE.57.1-2.53 (VISTA).

Loranger, J., Blonder, B., Garnier, E., Shipley, B., Vile, D., Violle, C. (2016). Occupancy and overlap in trait space along a successional gradient in Mediterranean old fields. American Journal of Botany, 103: 1050–1060. 10.3732/ajb.1500483 (INDIGO).

*Loranger, J., Violle, C., Shipley, B., Lavorel, S., Bonis, A., Cruz, P., Louault, F., Loucougaray, G., Mesléard, F., Yavercovski, N., et al. (2016). Recasting the dynamic equilibrium model through a functional lens: the interplay of trait-based community assembly and climate. Journal of Ecology, 104: 781–791. 10.1111/1365-2745.12536 (DivHerbe).

Navas, M.-L. Ducout, B., Roumet, C., et al. (2003). Leaf life span, dynamics and construction cost of species from Mediterranean old-fields differing in successional status. New Phytologist, 159 (1): 213-228. 10.1046/j.1469-8137.2003.00790.x (MELODY).

Navas, M.-L., Roumet, C., Bellmann, A., et al. (2010). Suites of plant traits in species from different stages of a Mediterranean secondary succession. Plant Biology, 12: 183-196. 10.1111/j.1438-8677.2009.00208.x (MELODY).

*Pakeman, R.J., Garnier, E., Lavorel, S., Ansquer, P., Castro, H., Cruz, P., Doležal, J. et al. (2008). Impact of abundance weighing on the response of seed traits to climate and land use. Journal of Ecology, 96: 355-366. 10.1111/j.1365-2745.2007.01336.x (VISTA).

*Pakeman, R.J., Lepš, J., Kleyer, M., Lavorel, S., Garnier, E., VISTA Consortium. (2009). Relative climatic, edaphic and management controls of plant functional trait signatures. Journal of Vegetation Science, 20: 148–159. 10.1111/j.1654-1103.2009.05548.x (VISTA).

Pérez-Ramos, I.M., Roumet, C., Cruz, P., Blanchard, A., Autran, P. & Garnier, E. (2012). Evidence for a « plant community economics spectrum » driven by nutrient and water limitations in a Mediterranean rangeland of Southern France. Journal of Ecology, 100: 1315-1327. 10.1111/1365-2745.12000 (DivHerbe).

Poirier V., Roumet C., Angers D.A., Munson A.D. (2018). Species and root traits impact macroaggregation in the rhizospheric soil of a Mediterranean common garden experiment. Plant and Soil, 424: 289-302. 10.1007/s11104-017-3407-6 (O2LA).

Prieto I, Birouste M., Zamora-Ledezma E., Gentit A., Goldin J., Volaire F., Roumet C. (2017). Decomposition rates of fine roots from three herbaceous perennial species: combined effect of root mixture composition and living plant community. Plant and Soil, 415: 359-372. 10.1007/s11104-016-3163-z (O2LA).

Prieto I., Querejeta J., Segrestin J., Volaire F., Roumet C. (2018). Leaf carbon and oxygen isotopes are coordinated with the leaf economics spectrum in Mediterranean rangeland species. Functional Ecology, 32: 612-625. 10.1111/1365-2435.13025 (O2LA).

Roumet C., Birouste M., Picon-Cochard C., Ghestem M., Osman N., Vrignon-Brenas S., Cao K., Stokes A. (2016). Root structure - function relationships in 74 herbaceous species: evidence of a root economics spectrum related to carbon economy. New Phytologist, 210: 815-826. 10.1111/nph.13828 (RESPIRS).

Segrestin, J., Bernard-Verdier, M., Violle, C., Richarte, J., Navas, M.-L., & Garnier, E. (2018). When is the best time to flower and disperse? A comparative analysis of plant reproductive phenology in the Mediterranean. Functional Ecology, 32: 1770-1783. 10.1111/1365-2435.13098 (PhenBiom).

Segrestin, J., Kazakou, E., Coq, S., Sartori, K., Richarte, J., Rowe, N.P. & Garnier, E. (2023). Responses of leaf biomechanics and underlying traits to rangeland management differ between graminoids and forbs. Journal of Vegetation Science, 34(6): e13216. 10.1111/jvs.13216 (PhenBiom).

Segrestin, J., Navas, M.-L., & Garnier, E. (2020). Reproductive phenology as a dimension of the phenotypic space in 139 plant species from the Mediterranean. New Phytologist, 225: 740-753. 10.1111/nph.16165 (PhenBiom).

Segrestin, J., Sartori, K., Navas, M.-L., Kattge, J., Díaz, S., Garnier, E. (2021). PhenoSpace: A Shiny application to visualize trait data in the phenotypic space of the global spectrum of plant form and function. Ecology and Evolution, ece3.6928. 10.1002/ece3.6928 (MELODY).

Shipley, B., Vile, D. & Garnier, E. (2006). From plant traits to plant communities: a statistical mechanistic approach to biodiversity. Science, 314: 812-814. 10.1126/science.1131344 (VISTA).

*Vile, D., Garnier, E., Shipley, B., Laurent, G., Navas, M.-L., Roumet, C., Lavorel, S., Díaz, S., Hodgson, J.G., Lloret, F., Midgley, G.F., Poorter, H., Rutherford, M.C., Wilson, P.J. & Wright, I.J. (2005). Specific leaf area and dry matter content estimate thickness in laminar leaves. Annals of Botany, 96: 1129-1136. 10.1093/aob/mci264 (DynEcoMed).

Vile, D., Shipley, B. & Garnier, E. (2006). A structural equation model to integrate changes in functional strategies during old-field succession. Ecology, 87: 504-517. 10.1890/05-0822 (VISTA).

Vile, D., Shipley, B. & Garnier, E. (2006). Ecosystem productivity can be predicted from potential relative growth rate and species abundance. Ecology Letters, 9: 1061-1067. 10.1111/j.1461-0248.2006.00958.x (VISTA).

Violle, C., Castro, H., Richarte, J., & Navas, M.-L. (2009). Intraspecific seed trait variations and competition: Passive or adaptive response? Functional Ecology, 23: 612–620. 10.1111/j.1365-2435.2009.01539.x (GEOTRAITS).

Violle C., Garnier E., Lecoeur J., Roumet C., Podeur C., Blanchard A., Navas M.-L. (2009). Competition, traits and resource depletion in plant communities. Oecologia, 160:747-755. 10.1007/s00442-009-1333-x (GEOTRAITS).

*Wright, I.J., Reich P.B., Cornelissen, J.H.C., Falster, D.S., Garnier, E., Hikosaka, K., Lamont, B.B., Lee, W., Oleksyn, J., Osada, N., Poorter, H., Villar, R., Warton, D.I., Westoby, M. (2005). Assessing the generality of global leaf trait relationships. New Phytologist, 166: 485-496. 10.1111/j.1469-8137.2005.01349.x (MELODY).

*Wright, I.J., Reich, P.B., Westoby, M., Ackerly, D.D., Baruch, Z., Bongers, F., Cavender-Bares, J. et al. (2004). The worldwide leaf economics spectrum. Nature, 428: 821-827. 10.1038/nature02403 (MELODY).

F. History of data set usage

1. **Data request history:** see publications with an "*" in front of the name of the first author in section Class V.B. ("Publications and results") above. For these large compilations and/or analyses, data aggregated at the species x treatment level were provided.
2. **Data set update history:** the version of FAIRTraits as described in this data paper is the first complete version of the database. It includes original data beyond those used in the publications listed in section Class V.B.

Acknowledgments

We thank Anabelle Dos Santos, Magdy El-Bana and the numerous students and technicians who contributed to the collection and processing of samples during the successive field campaigns throughout the period covered by the different projects. Many thanks to: James Aronson and Edouard Le Floc'h for creating favourable conditions for research at the Cazarils site; to Joël and Danièle Garnier for the re-birth of Les Agros; to the technical staff of the INRAE experimental farm at La Fage and of the CEFE experimental garden in Montpellier, a technical platform from the CeMEB LabEx (Centre Méditerranéen de l'Environnement et de la Biodiversité; ANR-10-LABX-0004 convention). Max Debussche and Colin Osborne consolidated the assignment of species to successional stages and photosynthetic pathways, respectively; Félix de Tombeur led us through the labyrinth of the WRB soil classification. The research projects which led to the initial collection of data are listed in Table 2 of this metadata document (section Class II.A.6.). This paper has benefited from the most welcome and enthusiastic assistance of Sophie Pamerlon (GBIF France), Yvan Le Bras and Olivier Norvez (both at the Pôle National des Données de la Biodiversité), in the context of the OpenMetaPaper project funded by the French Ministry of Higher Education and Research (FNSO project AAPFNSO2019OpenMetaPaper-14026).

Literature Citations

Anderegg, L. D. L. 2023. Why can't we predict traits from the environment? New Phytologist 237:1998–2004.

Aubin, I., F. Cardou, L. Boisvert-Marsh, E. Garnier, M. Strukelj, and A. D. Munson. 2020. Managing data locally to answer questions globally: the role of collaborative science in ecology. Journal of Vegetation Science 31:509–517.

Barkaoui, K., C. Roumet, and F. Volaire. 2016. Mean root trait more than root trait diversity determines drought resilience in native and cultivated Mediterranean grass mixtures. Agriculture, Ecosystems & Environment 231:122–132.

Bernard, C. 2008. Flore des Causses. Hautes terres, gorges, vallées et vallons (Aveyron, Lozère, Hérault et Gard). Bulletin de la Société Botanique du Centre-Ouest (Nouvelle série) 31:1–784.

de Bolòs, O., J. Vigo, R. Masalles, and J. Ninot. 1993. Flora manual dels països catalans. Second. Editorial Pòrtic s.a., Barcelona.

Braun-Blanquet, J., N. Roussine, and R. Nègre. 1952. Les Groupements Végétaux de la France Méditerranéenne. Centre National de la Recherche Scientifique, Montpellier.

Cornelissen, J. H. C., S. Lavorel, E. Garnier, S. Díaz, N. Buchmann, D. E. Gurvich, P. B. Reich, H. ter Steege, H. D. Morgan, M. G. A. van der Heijden, J. G. Pausas, and H. Poorter. 2003. A handbook of protocols for standardised and easy measurement of plant functional traits worldwide. Australian Journal of Botany 51:335–380.

Coste, A. H. 1937. Flore descriptive et illustrée de la France, de la Corse et des contrées limitrophes. Librairie des Sciences et des Arts, Paris, France.

Daget, P. 1977. Le bioclimat Méditerranéen: analyse des formes climatiques par le système d'Emberger. Vegetatio 34:87–103.

Delalandre, L. 2025. LDelalandre/FAIRTraits: V1.1 (try-release). Zenodo. https://doi.org/10.5281/zenodo.17073323

Ely, K. S., A. Rogers, D. A. Agarwal, E. A. Ainsworth, L. P. Albert, A. Ali, J. Anderson, M. J. Aspinwall, C. Bellasio, C. Bernacchi, S. Bonnage, T. N. Buckley, J. Bunce, A. C. Burnett, F. A. Busch, A. Cavanagh, L. A. Cernusak, R. Crystal-Ornelas, J. Damerow, K. J. Davidson, M. G. De Kauwe, M. C. Dietze, T. F. Domingues, M. E. Dusenge, D. S. Ellsworth, J. R. Evans, P. P. G. Gauthier, B. O. Gimenez, E. P. Gordon, C. M. Gough, A. H. Halbritter, D. T. Hanson, M. Heskel, J. A. Hogan, J. R. Hupp, K. Jardine, J. Kattge, T. Keenan, J. Kromdijk, D. P. Kumarathunge, J. Lamour, A. D. B. Leakey, D. S. LeBauer, Q. Li, M. R. Lundgren, N. McDowell, K. Meacham-Hensold, B. E. Medlyn, D. J. P. Moore, R. Negrón-Juárez, Ü. Niinemets, C. P. Osborne, A. L. Pivovaroff, H. Poorter, S. C. Reed, Y. Ryu, A. Sanz-Saez, S. C. Schmiege, S. P. Serbin, T. D. Sharkey, M. Slot, N. G. Smith, B. V. Sonawane, P. F. South, D. C. Souza, J. R. Stinziano, E. Stuart-Haëntjens, S. H. Taylor, M. D. Tejera, J. Uddling, V. Vandvik, C. Varadharajan, A. P. Walker, B. J. Walker, J. M. Warren, D. A. Way, B. T. Wolfe, J. Wu, S. D. Wullschleger, C. Xu, Z. Yan, and D. Yang. 2021. A reporting format for leaf-level gas exchange data and metadata. Ecological Informatics 61:101232.

Escarré, J., C. Houssard, and M. Debussche. 1983. Evolution de la végétation et du sol après abandon cultural en région méditerranéenne : étude de succession dans les garrigues du Montpelliérais (France). Acta Oecologica, Oecologia Plantarum 4:221–239.

Fort, F., F. Volaire, L. Guilioni, K. Barkaoui, M.-L. Navas, and C. Roumet. 2017. Root traits are related to plant water-use among rangeland Mediterranean species. Functional Ecology 31:1700–1709.

Freschet, G. T., L. Pagès, C. M. Iversen, L. H. Comas, B. Rewald, C. Roumet, J. Klimešová, M. Zadworny, H. Poorter, J. A. Postma, T. S. Adams, A. Bagniewska-Zadworna, A. G. Bengough, E. B. Blancaflor, I. Brunner, J. H. C. Cornelissen, E. Garnier, A. Gessler, S. E. Hobbie, I. C. Meier, L. Mommer, C. Picon-Cochard, L. Rose, P. Ryser, M. Scherer-Lorenzen, N. A. Soudzilovskaia, A.

Stokes, T. Sun, O. Valverde-Barrantes, M. Weemstra, A. Weigelt, N. Wurzburger, L. M. York, S. Batterman A., M. Gomes de Moraes, Š. Janeček, H. Lambers, V. Salmon, N. Tharayil, and M. L. McCormack. 2021. A starting guide to root ecology: strengthening ecological concepts and standardising root classification, sampling, processing and trait measurements. New Phytologist 232:973–1122.

Gallagher, R. V., D. S. Falster, B. S. Maitner, R. Salguero-Gómez, V. Vandvik, W. D. Pearse, F. D. Schneider, J. Kattge, J. H. Poelen, J. S. Madin, M. J. Ankenbrand, C. Penone, X. Feng, V. M. Adams, J. Alroy, S. C. Andrew, M. A. Balk, L. M. Bland, B. L. Boyle, C. H. Bravo-Avila, I. Brennan, A. J. R. Carthey, R. Catullo, B. R. Cavazos, D. A. Conde, S. L. Chown, B. Fadrique, H. Gibb, A. H. Halbritter, J. Hammock, J. A. Hogan, H. Holewa, M. Hope, C. M. Iversen, M. Jochum, M. Kearney, A. Keller, P. Mabee, P. Manning, L. McCormack, S. T. Michaletz, D. S. Park, T. M. Perez, S. Pineda-Munoz, C. A. Ray, M. Rossetto, H. Sauquet, B. Sparrow, M. J. Spasojevic, R. J. Telford, J. A. Tobias, C. Violle, R. Walls, K. C. B. Weiss, M. Westoby, I. J. Wright, and B. J. Enquist. 2020. Open Science principles for accelerating trait-based science across the Tree of Life. Nature Ecology & Evolution 4:294-303.

Garnier, E., J. Cortez, G. Billès, M.-L. Navas, C. Roumet, M. Debussche, G. Laurent, A. Blanchard, D. Aubry, A. Bellmann, C. Neill, and J.-P. Toussaint. 2004. Plant functional markers capture ecosystem properties during secondary succession. Ecology 85:2630–2637.

Garnier, E., G. Laurent, A. Bellmann, S. Debain, P. Berthelier, B. Ducout, C. Roumet, and M.-L. Navas. 2001. Consistency of species ranking based on functional leaf traits. New Phytologist 152:69–83.

Garnier, E., S. Lavorel, P. Ansquer, H. Castro, P. Cruz, J. Dolezal, O. Eriksson, C. Fortunel, H. Freitas, C. Golodets, K. Grigulis, C. Jouany, E. Kazakou, J. Kigel, M. Kleyer, V. Lehsten, J. Lepš, T. Meier, R. J. Pakeman, M. Papadimitriou, V. . P. Papanastasis, H. Quested, F. Quétier, M. Robson, C. Roumet, G. Rusch, M. Skarpe, M. Sternberg, J.-P. Theau, A. Thébault, D. Vile, and M. Zarovali. 2007. Assessing the effects of land use change on plant traits, communities and ecosystem functioning in grasslands: a standardized methodology and lessons from an application to 11 European sites. Annals of Botany 99:967–985.

Garnier, E., M.-L. Navas, and K. Grigulis. 2016. Plant Functional Diversity - Organism Traits, Community Structure, and Ecosystem Properties. Oxford University Press, Oxford.

Garnier, E., U. Stahl, M.-A. Laporte, J. Kattge, I. Mougenot, I. Kühn, B. Laporte, B. Amiaud, F. S. Ahrestani, G. Bönisch, D. E. Bunker, J. H. C. Cornelissen, S. Díaz, B. J. Enquist, S. Gachet, P. Jaureguiberry, M. Kleyer, S. Lavorel, L. Maicher, N. Pérez-Harguindeguy, H. Poorter, M. Schildhauer, B. Shipley, C. Violle, E. Weiher, C. Wirth, I. J. Wright, and S. Klotz. 2017. Towards a thesaurus of plant characteristics: an ecological contribution. Journal of Ecology 105:298–309.

Hampton, S. E., S. S. Anderson, S. C. Bagby, C. Gries, X. Han, E. M. Hart, M. B. Jones, W. C. Lenhardt, A. MacDonald, W. K. Michener, J. Mudge, A. Pourmokhtarian, M. P. Schildhauer, K. H. Woo, and N. Zimmerman. 2015. The Tao of open science for ecology. Ecosphere 6:1–13.

Kattge, J., G. Bönisch, S. Díaz et al. 2020. TRY plant trait database – enhanced coverage and open access. Global Change Biology 26:119–188.

Kazakou, E., E. Garnier, M.-L. Navas, C. Roumet, C. Collin, and G. Laurent. 2007. Components of nutrient residence time and the leaf economics spectrum in species from Mediterranean old-fields differing in successional status. Functional Ecology 21:235–245.

Keller, A., M. J. Ankenbrand, H. Bruelheide, S. Dekeyzer, B. J. Enquist, M. B. Erfanian, D. S. Falster, R. V. Gallagher, J. Hammock, J. Kattge, S. D. Leonhardt, J. S. Madin, B. Maitner, M. Neyret, R. E. Onstein, W. D. Pearse, J. H. Poelen, R. Salguero-Gomez, F. D. Schneider, A. B. Tóth, and C. Penone. 2023. Ten (mostly) simple rules to future-proof trait data in ecological and evolutionary sciences. Methods in Ecology and Evolution 14:444–458.

Komsta, L. 2022. outliers: Tests for Outliers. R package version 0.15, https://CRAN.R-project.org/package=outliers.

Le Floc'h, E., J. Aronson, S. Dhillion, J.-L. Guillerm, A. Grossmann, and E. Cunge. 1998. Biodiversity and ecosystem trajectories: first results from a new LTER in southern France. Acta Oecologica 19:285–293.

Lloret, F., and M. Vilá. 2003. Diversity patterns of plant functional types in relation to fire regime and previous land use in Mediterranean woodlands. Journal of Vegetation Science 14:387–398.

Maechler, M. 2024. diptest: Hartigan's Dip Test Statistic for Unimodality - Corrected. R package version 0.77-1, https://CRAN.R-project.org/package=diptest.

McCormack, M. L., D. Guo, C. M. Iversen, W. Chen, D. M. Eissenstat, C. W. Fernandez, L. Li, C. Ma, Z. Ma, H. Poorter, et al. 2017. Building a better foundation: improving root-trait measurements to understand and model plant and ecosystem processes. New Phytologist 215:27–37.

Michener, W. K., J. W. Brunt, J. J. Helly, T. B. Kirchner, and S. G. Stafford. 1997. Nongeospatial metadata for the ecological sciences. Ecological Applications 7:330–342.

Molénat, G., D. Foulquié, P. Autran, J. Bouix, D. Hubert, M. Jacquin, F. Bocquier, and B. Bibé. 2005. Pour un élevage ovin allaitant performant et durable sur parcours: un système expérimental sur le Causse du Larzac. INRA Productions Animales 18:323–338.

Mungall, C. J., G. V. Gkoutos, C. L. Smith, M. A. Haendel, S. E. Lewis, and M. Ashburner. 2010. Integrating phenotype ontologies across multiple species. Genome Biology 11:R2.

Navas, M.-L., C. Roumet, A. Bellmann, G. Laurent, and E. Garnier. 2010. Suites of plant traits in species from different stages of a Mediterranean secondary succession. Plant Biology 12:183–196.

Pakeman, R. J., and Quested, H. M. 2007. Sampling plant functional traits: What proportion of the species need to be measured? Applied Vegetation Science 10: 91–96.

Parr, C. S., N. Wilson, P. Leary, K. Schulz, K. Lans, L. Walley, J. Hammock, A. Goddard, J. Rice, M. Studer, J. Holmes, and R. Corrigan, Jr. 2014. The Encyclopedia of Life v2: Providing global access to knowledge about life on Earth. Biodiversity Data Journal 2:e1079.

Poisot, T., A. Bruneau, A. Gonzalez, D. Gravel, and P. Peres-Neto. 2019. Ecological data should not be so hard to find and reuse. Trends in Ecology & Evolution 34:494–496.

R Core Team 2025. R: A Language and Environment for Statistical Computing. R Foundation for Statistical Computing, Vienna, Austria. https://www.R-project.org/.

Raunkiaer, C. 1934. The Life Forms of Plants and Statistical Plant Geography. Page (H. Gilbert-Carter, Tran.). English Edition. Oxford University Press, Oxford.

Schneider, F. D., D. Fichtmueller, M. M. Gossner, A. Güntsch, M. Jochum, B. König-Ries, G. Le Provost, P. Manning, A. Ostrowski, C. Penone, and N. K. Simons. 2019. Towards an ecological trait-data standard. Methods in Ecology and Evolution 10:2006–2019.

Shipley, B., F. De Bello, J. H. C. Cornelissen, E. Laliberté, D. C. Laughlin, and P. B. Reich. 2016. Reinforcing loose foundation stones in trait-based plant ecology. Oecologia 180:923–931.

Tavşanoğlu, Ç., and J. G. Pausas. 2018. A functional trait database for Mediterranean Basin plants. Scientific Data 5:180135.

Tison, J.-M., P. Jauzein, H. Michaud, D. Jeanmono, and F. Boillot. 2014. Flore de la France méditerranéenne continentale. 1ère édition. Naturalia Publications, Turriers Porquerolles.

Violle, C., M.-L. Navas, D. Vile, E. Kazakou, C. Fortunel, I. Hummel, and E. Garnier. 2007. Let the concept of trait be functional! Oikos 116:882–892.

Walter, H., E. Harnickell, and D. Mueller-Dombois. 1975. Climate-diagram maps of the individual continents and the ecological climatic regions of the Earth. Springer-Verlag, Berlin.

Wieczorek, J., D. Bloom, R. Guralnick, S. Blum, M. Doring, R. Giovanni, T. Robertson, and D. Vieglais. 2012. Darwin Core: An evolving community-developed biodiversity data standard. PLoS ONE 7.

Wilkinson, M. D., M. Dumontier, Ij. J. Aalbersberg, G. Appleton, M. Axton, A. Baak, N. Blomberg, J.-W. Boiten, L. B. da Silva Santos, P. E. Bourne, J. Bouwman, A. J. Brookes, T. Clark, M. Crosas, I. Dillo, O. Dumon, S. Edmunds, C. T. Evelo, R. Finkers, A. Gonzalez-Beltran, A. J. G. Gray, P. Groth, C. Goble, J. S. Grethe, J. Heringa, P. A. C. 't Hoen, R. Hooft, T. Kuhn, R. Kok, J. Kok, S. J. Lusher, M. E. Martone, A. Mons, A. L. Packer, B. Persson, P. Rocca-Serra, M. Roos, R. van Schaik, S.-A. Sansone, E. Schultes, T. Sengstag, T. Slater, G. Strawn, M. A. Swertz, M. Thompson, J. van der Lei, E. van Mulligen, J. Velterop, A. Waagmeester, P. Wittenburg, K. Wolstencroft, J. Zhao, and B. Mons.

2016. The FAIR Guiding Principles for scientific data management and stewardship. Scientific Data 3:160018.

WRB. 2022. World Reference Base for Soil Resources. IUSS Working Group WRB 2022. ISBN 979-8-9862451-1-9
